# Survival Patterns Among Patients With Breast Cancer in Sub-Saharan Africa: A Systematic Review and Meta-Analysis

**DOI:** 10.1001/jamanetworkopen.2024.10260

**Published:** 2024-05-14

**Authors:** Miteku Andualem Limenih, Eskedar Getie Mekonnen, Frehiwot Birhanu, Beshada Rago Jima, Binyam Girma Sisay, Eskeziaw Abebe Kassahun, Hamid Yimam Hassen

**Affiliations:** 1 Department of Epidemiology and Biostatistics, Institute of Public Health, College of Medicine and Health Sciences, University of Gondar, Gondar, Ethiopia; 2 Department of Family Medicine and Population Health, Faculty of Medicine and Health Sciences, University of Antwerp, Antwerp, Belgium; 3 Department of Health Service Management, School of Public Health, College of Health Science, Mizan-Tepi University, Mizan-Aman, Ethiopia; 4 Department of Nutrition and Dietetics, School of Public Health, Addis Ababa University, Addis Ababa, Ethiopia; 5 School of Exercise and Nutritional Sciences, Faculty of Health, Deakin University, Melbourne, Victoria, Australia; 6 VITO Health, Flemish Institute for Technological Research (VITO), Mol, Belgium

## Abstract

**Importance:**

Breast cancer is the most prevalent cancer globally with tremendous disparities both within specific regions and across different contexts. The survival pattern of patients with breast cancer remains poorly understood in sub-Saharan African (SSA) countries.

**Objective:**

To investigate the survival patterns of patients with breast cancer in SSA countries and compare the variation across countries and over time.

**Data Sources:**

Embase, PubMed, Web of Science, Scopus, and ProQuest were searched from inception to December 31, 2022, with a manual search of the references.

**Study Selection:**

Cohort studies of human participants that reported 1-, 2-, 3-, 4-, 5-, and 10-year survival from diagnosis among men, women, or both with breast cancer in SSA were included.

**Data Extraction and Synthesis:**

Independent extraction of study characteristics by multiple observers was performed using open-source software, then exported to a standard spreadsheet. A random-effects model using the generalized linear mixed-effects model was used to pool data. The Preferred Reporting Items for Systematic Review and Meta-Analyses (PRISMA) guideline for reporting was followed.

**Main Outcome and Measures:**

Survival time from diagnosis.

**Results:**

Forty-nine studies were included in the review with a sample size ranging from 21 to 2311 (total, 14 459; 196 [1.35%] men, 13 556 [93.75%] women, and 707 [4.90%] unspecified; mean age range, 38 to 71 years), of which 40 were summarized using meta-analysis. The pooled 1-year survival rate of patients with breast cancer in SSA was 0.79 (95% CI, 0.67-0.88); 2-year survival rate, 0.70 (95% CI, 0.57-0.80); 3-year survival rate, 0.56 (95% CI, 0.45-0.67); 4-year survival rate, 0.54 (95% CI, 0.43-0.65); and 5-year survival rate, 0.40 (95% CI, 0.32-0.49). The subgroup analysis showed that the 5-year survival rate ranged from 0.26 (95% CI, 0.06-0.65) for studies conducted earlier than 2010 to 0.47 (95% CI, 0.32-0.64) for studies conducted later than 2020. Additionally, the 5-year survival rate was lower in countries with a low human development index (HDI) (0.36 [95% CI, 0.25-0.49) compared with a middle HDI (0.46 [95% CI, 0.33-0.60]) and a high HDI (0.54 [95% CI, 0.04-0.97]).

**Conclusions and Relevance:**

In this systematic review and meta-analysis, the survival rates for patients with breast cancer in SSA were higher in countries with a high HDI compared with a low HDI. Enhancing patient survival necessitates a comprehensive approach that involves collaboration from all relevant stakeholders.

## Key Points

**Question:**

What is the pattern of survival for patients with breast cancer in Sub-Saharan African countries?

**Findings:**

In this systematic review and meta-analysis of 14 459 participants in 49 unique studies, the survival rates for patients with breast cancer were estimated to be 79% at 1 year, 56% at 3 years, and 40% at 5 years. The survival was lower in countries with a low compared with middle and high human development indexes, and an improvement in survival was observed in studies conducted in recent years.

**Meaning:**

These findings suggest that a comprehensive approach—including breast cancer screening, early diagnosis, and effective treatment—involving collaboration from all relevant stakeholders is necessary to enhance the lower survival rates of breast cancer in sub-Saharan Africa.

## Teaser

This systematic review and meta-analysis estimates the survival pattern of patients with breast cancer in Sub-Saharan African countries and explores variations across countries, over time and by development status.

## Introduction

The overall morbidity and mortality associated with breast cancer have been steadily increasing in the past 3 decades.^1^ Since 1990, the global annual percentage change for breast cancer mortality increased by 0.23%.^2^ As of the end of 2020, breast cancer–related disability-adjusted life-years increased to 20 625 313, making it the world’s most prevalent cancer.^3^ Moreover, breast cancer accounts for 1 in 8 cancer diagnoses and 1 in 6 cancer-related deaths, establishing it as the leading cause of cancer-related mortality among women worldwide.^4^ However, a significant disparity in breast cancer incidence and mortality exists across different regions of the world.

The annual increase in the incidence of breast cancer depends, in part, on the country’s human development index (HDI).^5^ Countries with a high HDI, such as the US and European countries, had an annual breast cancer incidence rate increase of less than 0.5% (age-standardized incidence rate, 75.6 cases/100 000 population), whereas low- and middle-HDI regions including sub-Saharan African (SSA) countries experienced an increase in age-standardized incidence rate of greater than 5% (low-HDI countries, 27.8 cases/100 000; middle-HDI countries, 36.1 cases/100 000).^2^ On the other hand, breast cancer mortality is higher in countries with low or medium HDI (age-standardized mortality rates, 17.1 and 14.3 cases/100 000, respectively) compared with countries with a high HDI (10.3 cases/100 000).^6^

While high-income countries have made significant progress in reducing age-standardized breast cancer mortality by 40% over the past 4 decades, equivalent to an annual reduction of 2% to 4%,^7^ low- and middle-income countries (LMIC) face challenges, as they have the highest mortality-to-incidence ratio (MIR), indicating a less responsive health care system and a significant disease burden. The MIR in LMIC stands at 0.55, while it was 0.16 in high-income countries.^8^ Several factors could contribute to the observed difference in MIR of breast cancer, including late-stage disease presentation, inadequate diagnostic and treatment facilities, and limited access to high-quality comprehensive health care services in LMIC.^9,10^

The incidence and mortality rates of breast cancer also vary across and within countries and regions in Africa. Southern Africa has the highest incidence rate (46.2 cases/100 000), followed by Western (37.3 cases/100 000) and Eastern Africa (29.9 cases/100 000). In contrast, Western Africa has the highest mortality rate (18.9 cases/100 000).^11^ The higher mortality in the region can be attributed to suboptimal screening and therapeutic infrastructure due to limited resources, as many of the world’s poorest countries are in this region.^12^ Additionally, individual-level factors such as low awareness of risk factors and delayed health-seeking practices contribute to lower breast cancer survival.^13,14^

In 2021, the World Health Organization initiated the “Global Breast Cancer Initiative” to reduce global breast cancer mortality rates by 2.5% annually by uniting stakeholders worldwide toward this common goal.^15^ An essential aspect of this initiative is the estimation of breast cancer incidence and mortality, which informs policies and practices. However, nearly half (46.6%) of the World Health Organization member states in SSA lack cancer registries, hindering the accurate assessment of disease and economic burdens in each country. This absence of reliable data makes it challenging to monitor progress in breast cancer diagnosis and treatment in SSA. Thus, understanding the breast cancer survival patterns in SSA countries, where it remains poorly understood, is crucial. Therefore, this study aimed to estimate the survival pattern of breast cancer in SSA countries and explore variations across countries, over time and by development status.

## Methods

The protocol of this review is registered in the PROSPERO International Prospective Register of systematic reviews (CRD42022339173). The review adhered to the Preferred Reporting Items for Systematic Review and Meta-Analyses (PRISMA) 2020 guideline.

### Information Source and Search Strategy

A thorough search was conducted using relevant medical subject headings in databases including Embase, PubMed, Web of Science, Scopus, and ProQuest. Articles from inception to December 31, 2022, were retrieved. Additional databases and reference lists were also consulted for potential studies (eTable 1 in Supplement 1).

### Eligibility Criteria

The review included all cohort studies in human participants that reported breast cancer survival among men, women, or both in SSA. The study population included patients diagnosed with breast cancer, and the primary outcome was survival time (1, 2, 3, 4, 5, and 10 years) from diagnosis; moreover, overall mortality was also considered.

### Study Screening

Articles from both the database and manual searches were exported into an EndNote library, version 9 (Clarivate), where duplicate articles were identified and subsequently removed. Nonduplicate articles were then imported into a web-based artificial intelligence–based tool, Rayyan.QCRI.org.^16^ All retrieved titles and abstracts underwent a double-screening process using predefined criteria. Further full-text screening was performed for the titles and abstracts that met the inclusion criteria to determine final eligibility for inclusion.

### Data Extraction

Muiltiple reviewers working in pairs (M.A.L. and F.B., E.G.M. and H.Y.H., and B.R.S. and B.G.S.) extracted all pertinent information, including the survival measure with its associated 95% CI, study design, characteristics of the study participants, quality assessment, how outcomes were ascertained, and general information about the articles. We did not include racial and ethnic data due to most individuals in the study being homogeneous, and most studies did not incorporate such information. Consequently, inclusion of these data was not feasible. To facilitate the data extraction, a tool was prepared using open-source Kobo Toolbox. The extracted data were subsequently exported to an Excel spreadsheet, version 2108 (Microsoft Corp), for further processing and analysis. In cases where the results were only presented graphically (eg, Kaplan-Meier curves) and could not be obtained directly from the corresponding authors, the relevant information was extracted using WebPlotDigitizer (automeris.io), a tool known for its capacity to provide valid estimates.^17^

### Quality Assessment and Critical Appraisal

The Newcastle-Ottawa Quality Assessment Scale for cohort studies was used. The quality measures were divided into 3 domains: selection (D1), comparability (D2), and outcome measure (D3). Considering the study, we provided a higher weight for D3. The overall risk bias was coded as high if D3 was poor and if the study had a poor score for both D1 and D2 but a fair score for D3; moderate risk of bias if D1 and D2 had poor scores but D3 had a good score; and low risk of bias if all domains had good scores or if the risk of bias was high for D1 or D2 only.

### Statistical Analysis

Descriptive statistics were used to summarize study characteristics. The meta-analysis was performed at various time points (1, 2, 3, 4, 5, and 10 years) to account for variations in follow-up duration and frequency of survival status across studies. A random-effects model using the generalized linear mixed model was used in the analysis. We quantified between-study heterogeneity using *I*^2^ statistics, and the significance of heterogeneity was tested with the Cochran Q test. Publication bias was assessed graphically using funnel plots, and the statistical significance was tested using the Egger regression.^18^

Subgroup analysis was based on the study period and the country’s HDI. The country’s HDI was categorized based on the HDI rank of the country in 2021.^19^ Furthermore, meta-regression was performed to examine the association of study-level covariates with breast cancer survival in SSA.

Data were analyzed using the free statistical software R, version 4.3.1 (R Project for Statistical Computing). Different packages were used, including survival, survminer, metafor, and rmeta. A 2-sided test was used for all hypotheses with a significance level of 5% (*P* < .05) and reporting the corresponding 95% CI.

## Results

### Description of the Studies

**Figure 1.**
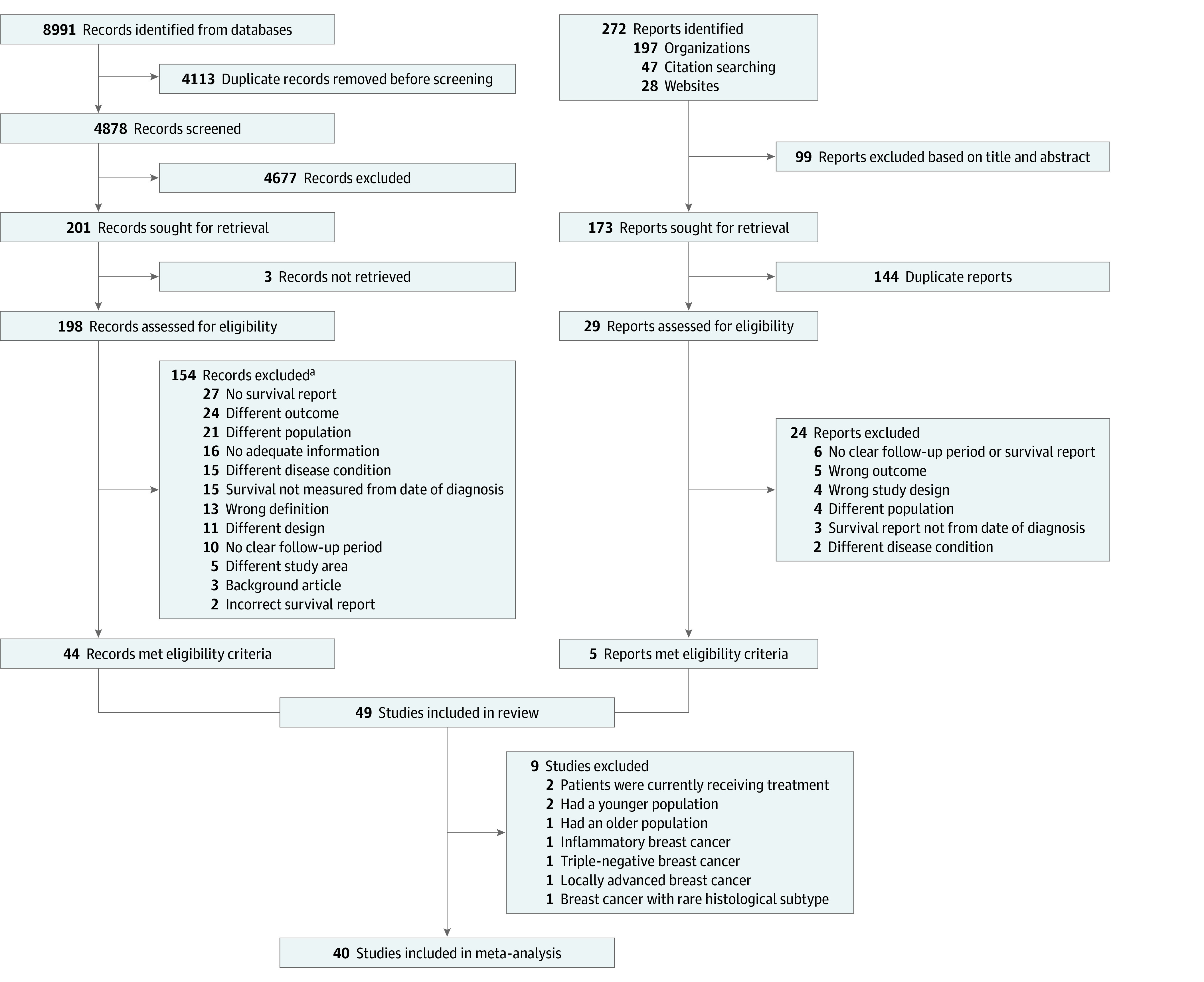
Study Flow Diagram of the Article Search and Selection Process ^a^Eight records were excluded for more than 1 reason.

Our initial database search yielded a total of 8991 abstracts. After removing duplicates, 7437 articles underwent abstract title screening. This screening process led to the selection of 190 articles. Through searches of references, websites, and organizational registers, we identified 11 more articles, resulting in a total of 201 articles. Following a thorough full-text review, 69 studies were reviewed. Of these, 49 studies that met the eligibility criteria^20,21,22,23,24,25,26,27,28,29,30,31,32,33,34,35,36,37,38,39,40,41,42,43,44,45,46,47,48,49,50,51,52,53,54,55,56,57,58,59,60,61,62,63,64,65,66,67,68^ were included in the narrative synthesis, and 40 of them^20,21,22,23,25,27,29,30,31,32,33,34,35,36,38,39,41,42,43,45,46,47,48,49,50,51,52,53,54,55,56,57,58,61,62,63,64,65,66,68^ underwent meta-analysis for a more in-depth and comprehensive analysis (Figure 1).

### Study Characteristics

Of 49 studies included in the systematic review, 13 were conducted in Nigeria^20,21,22,23,24,25,26,27,28,29,30,31,32^; 4 each in Ethiopia^33,34,35,36^ and South Africa^37,38,39,40^; 3 each in Sudan,^41,42,43^ Ghana,^44,45,46^ Uganda,^47,48,49^ and Burkina Faso^50,51,52^; 2 each in Kenya,^53,54^ Malawi,^55,56^ Cameroon,^57,58^ Senegal,^59,60^ and multiple countries^61,62^; and 1 each in Gambia,^63^ Zimbabwe,^64^ Tanzania,^65^ Mozambique,^66^ Congo,^67^ and Guinea.^68^ More than two-thirds of the studies were published after 2010. Thirty-one studies (63.3%) included only female participants,^20,25,26,27,28,33,34,36,37,38,39,40,41,42,45,46,48,50,51,53,54,55,56,57,58,59,60,61,62,66,67^ while the remaining studies included both female and male (30.6%)^23,24,29,30,31,32,35,43,44,47,49,63,64,65,68^ or only male (6.1%) participants.^21,22,52^ The proportion of female participants was higher in the studies that included both sexes. Most of the studies recruited participants with all stages of breast cancer to calculate overall survival.

The sample size of the included studies ranged from 21 to 2311 (total, 14 459; 196 [1.35%] men, 13 556 [93.75%] women, and 707 [4.90%] unspecified). Minimum and maximum mean ages of participants were 38 and 71, respectively. Studies used various methods of death ascertainment, including medical records, death certificates, verbal autopsy, and a combination of any of these. Details of the study characteristics are presented in eTable 2 in Supplement 1.

Nine studies were excluded from the meta-analysis due to their inclusion of specific populations such as younger populations,^37,60^ older populations,^40^ and patients with inflammatory breast cancer,^67^ triple-negative breast cancer,^59^ locally advanced breast cancer,^24^ and breast cancer with rare histological subtypes.^28^ Studies only conducted among patients currently receiving treatment^26,44^ were also excluded from the meta-analysis.

Based on the Newcastle-Ottawa Quality Assessment scale, 26 studies had low, 2 studies had moderate, and 21 studies had high risk of bias. A summary of the assessment for individual studies in each domain is available in eFigure 1 in Supplement 1.

### Meta-Analysis

**Figure 2.**
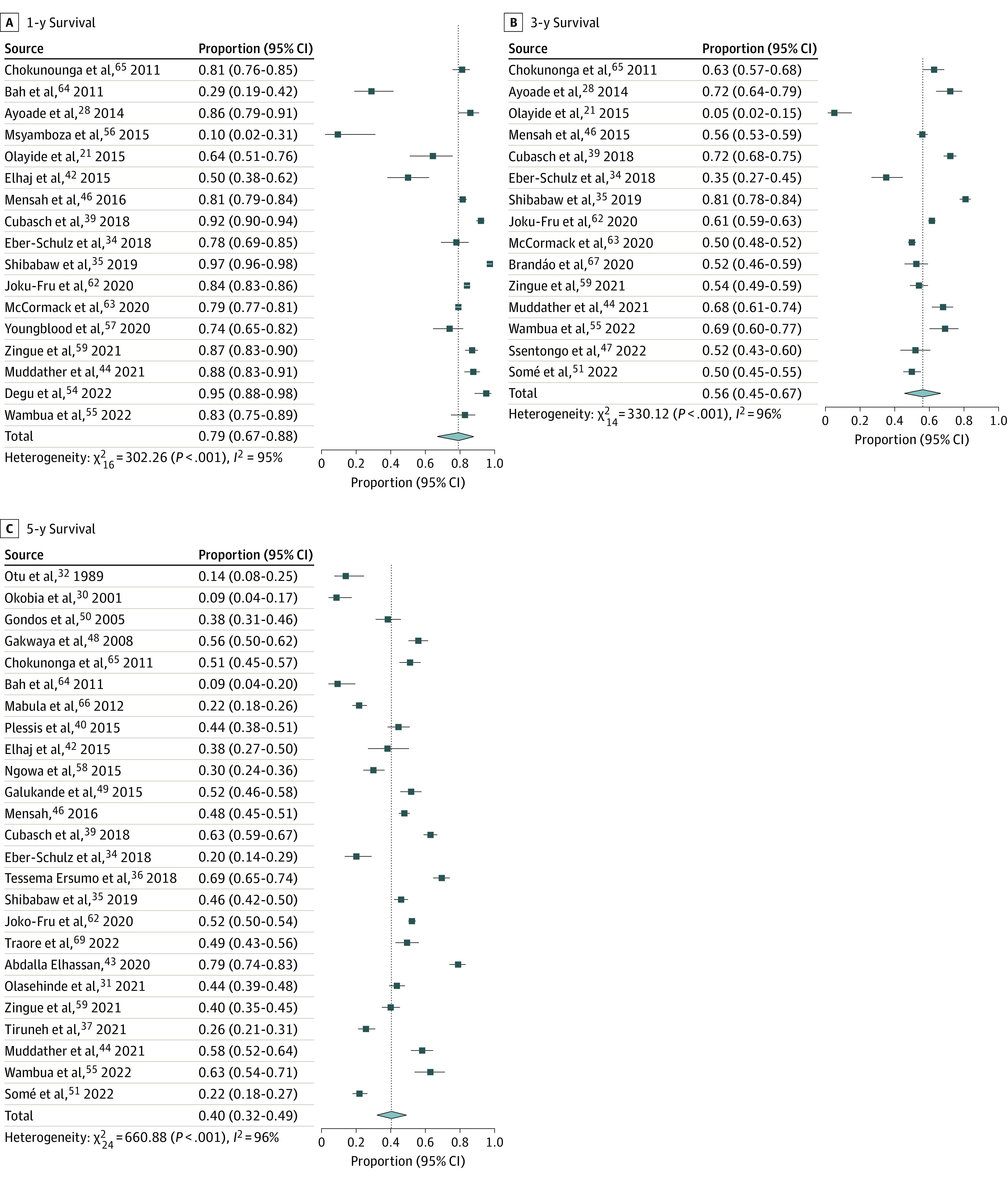
Survival Rates Among Patients With Breast Cancer in Sub-Saharan Africa Boxes indicate proportions; error bars indicate 95% CIs. Diamonds indicate pooled estimates.

The 1-year survival rate was 0.79 (95% CI, 0.67-0.88) estimated from 17 studies. A meta-analysis of 15 studies showed a 3-year survival rate of 0.56 (95% CI, 0.45-0.67), and the pooled 5-year survival rate, based on data from 25 studies, was 0.40 (95% CI, 0.32-0.49) (Table 1). The 1-, 3- and 5-year survival rates are shown in Figure 2, while the forest plots for 2-year (0.70 [95% CI, 0.57-0.80]) and 4-year (0.54 [95% CI, 0.43-0.65]) survival rates can be found in eFigure 2 in Supplement 1. Moreover, based on 3 studies, 5-year survival among male participants with breast cancer was 0.31 (95% CI, 0.09-0.67) (eFigure 3 in Supplement 1).

**Table 1.** Pooled Survival Rates of Patients With Breast Cancer in Sub-Saharan Africa

Survival	No. of studies	Total No. of patients	Pooled proportion (95% CI)	*I*^2^ (95% CI), %
1-y	17	8308	0.79 (0.67-0.88)	95 (93-96)
2-y	11	3835	0.70 (0.57-0.80)	96 (95-97)
3-y	15	8670	0.56 (0.45-0.67)	96 (94-97)
4-y	8	3182	0.54 (0.43-0.65)	94 (91-96)
5-y	25	9481	0.40 (0.32-0.49)	96 (95-97)

### Heterogeneity

Considerable heterogeneity was observed across studies in 1-year (*I*^2^ = 95% [95% CI, 93%-96%]), 2-year (*I*^2^ = 96% [95% CI, 95%-97%]), 3-year (*I*^2^ = 96% [95% CI, 94%-97%]), 4-year (*I*^2^ = 94% [95% CI, 91%-96%]), and 5-year (*I*^2^ = 96% [95% CI, 95%-97%]) survival rates. Results of the χ^2^ test showed that the observed heterogeneity for all time points was statistically significant (*P* < .001).

### Subgroup Analysis and Meta-Regression

The results of the subgroup analysis revealed variation in the pooled survival rate across study periods and the country’s HDI category (Table 2). The 1-year survival rate varied across study periods, with the highest rate observed in the most recent period, 2020 or after (0.85 [95% CI, 0.77-0.91]), compared with 2015 to 2019 (0.83 [95% CI, 0.64-0.93]) and 2010 to 2014 (0.52 [95% CI, 0.07-0.94]). However, these subgroup differences were not statistically significant (*P* = .16). Likewise, the 5-year survival rate ranged from 0.26 (95% CI, 0.06-0.65) for the period earlier than 2010 to 0.47 (95 CI%, 0.32-0.64) for the period later than 2020 (*P* = .14). Subgroup analysis based on HDI showed that the 1-year survival was lower in low-HDI countries (0.70 [95% CI, 0.41-0.88]) than in middle-HDI (0.85 [95% CI, 0.78-0.91]) and high-HDI (0.92 [95% CI, 0.90-0.94]) countries, and these subgroup differences were statistically significant (*P* < .001). A similar pattern was observed in the 5-year survival rate, showing a lower survival rate in low-HDI countries (0.36 [95% CI, 0.25-0.49]) than in middle-HDI (0.46 [95% CI, 0.33-0.60]) and high-HDI (0.54 [95% CI, 0.04-0.97]) countries (*P* = .04) (eFigure 4 in Supplement 1).

**Table 2.** Subgroup Analysis of Survival of Patients With Breast Cancer in Sub-Saharan Africa

Subgroup	1-y Data		3-y Data		5-y Data	
	No. of studies	Survival (95% CI)	No. of studies	Survival (95% CI)	No. of studies	Survival (95% CI)
Study period						
Before 2010	NA	NA	NA	NA	4	0.26 (0.06- 0.65)
2010 to 2014	4	0.52 (0.07-0.94)	2	0.67 (0.23-0.93)	3	0.24 (0.03-0.78)
2015 to 2019	7	0.83 (0.64-0.93)	6	0.50 (0.20-0.80)	10	0.46 (0.36-0.57)
2020 and later	6	0.85 (0.77-0.91)	7	0.56 (0.49-0.63)	8	0.47 (0.32-0.64)
*P* value	NA	.16	NA	.05	NA	.14
HDI						
Low	9	0.70 (0.41-0.88)	7	0.50 (0.24-0.76)	17	0.36 (0.25-0.49)
Middle	5	0.85 (0.78-0.91)	5	0.58 (0.51-0.65)	5	0.46 (0.33-0.60)
High	1	0.92 (0.90-0.94)	1	0.72 (0.68-0.75)	2	0.54 (0.04-0.97)
Multiple countries	2	0.82 (0.49-0.95)	2	0.56 (0.14-0.91)	1	0.52 (0.50-0.54)
*P* value	NA	<.001	NA	<.001	NA	.04
Study quality						
Good	10	0.87 (0.80-0.91)	11	0.63 (0.55-0.69)	14	0.47 (0.36-0.59)
Fair	1	0.29 (0.19-0.42)	4	0.35 (0.06-0.82)	2	0.21 (0.00-1.00)
Poor	6	0.68 (0.29-0.91)	NA	NA	9	0.35 (0.23-0.49)
*P* value	NA	<.001	NA	.09	NA	.11

Abbreviations: HDI, human development index; NA, not applicable.

The results of the meta-regression are summarized in Table 3. The study level covariates included in the meta-regression were study year, sample size, study quality, and country’s HDI category. The results showed that the study year was associated with the pooled 1-year survival rate estimate (adjusted β, 0.120 [95% CI, 0.06-0.34]; *P* < .001), with a better survival rate observed in more recent studies. Study quality was also associated with pooled 1-year survival rate estimates after controlling for the country’s HDI and the study period (adjusted β, −0.64 [95% CI, −1.19 to −0.09]; *P* = .03), indicating better survival rate in higher quality studies. However, it should be noted that the number of studies in each category of study quality was unbalanced. Specifically, for 1-year survival, only 1 study was categorized as fair quality, which reported an exceptionally lower survival rate (0.29) compared with studies categorized as poor quality (0.68) and good quality (0.87). Overall, study level covariates included in the meta-regression explained 72% of the between-study variability in the 1-year survival estimate (*R*^2^ = 71.7% [*P* = .02]). In addition, the study year was also associated with the pooled 5-year survival estimate (adjusted β, 0.05 [95% CI, 0.01-0.10]; *P* = .02), with better survival in recent years.

**Table 3.** Multivariable Meta-Regression of Breast Cancer Survival in Sub-Saharan Africa

Variable	Adjusted β (95% CI)		
	1-y Survival	3-y Survival	5-y Survival
Study year	0.20 (0.06 to 0.34)^a^	0.06 (−0.07 to 0.18)	0.05 (0.01 to 0.10)^b^
Sample size	−0.001 (−0.002 to 0.001)	−0.0001 (−0.001 to 0.001)	−0.0001 (−0.001 to 0.001)
Study quality	−0.64 (−1.19 to −0.09)^b^	−0.58 (−1.06 to −0.09)^b^	−0.15 (−0.51 to 0.20)
Country’s HDI	0.22 (−0.68 to 1.11)	0.002 (−0.67 to 0.68)	0.19 (−0.31 to 0.70)
Overall test of moderators, *P* value	.02	.19	.06
*R*^2^ value, %	71.7	40.1	33.1

Abbreviation: HDI, human development index.

^a^ *P* < .01.

^b^ *P* < .05.

### Publication Bias

Funnel plots of study size against log odds indicated no major issue of publication bias for all the pooled survival estimates. This finding was also supported by the results of the Egger test of symmetry (eFigure 5 in Supplement 1).

## Discussion

The results of this meta-analysis indicate that the estimated survival rates were 79% at 1 year, 56% at 3 years, and 40% at 5 years. Notably, the 5-year survival among men was 31%. The subgroup analysis indicated that better survival was observed in higher-HDI countries and more recent studies.

The overall survival rate of patients with breast cancer in this study was much lower than that of a globally conducted systematic review by Maajani et al,^69^ in which the pooled survival rates across 130 studies were 92% for 1 year, 75% for 3 years, and 73% for 5 years. The observed variation in breast cancer survival rates could be explained by each country’s HDI as indicated by the study. The HDI, which considers factors such as life expectancy, educational level, and income, reflects the overall development and resources available within a country.^19^ Lower HDI scores in certain African countries may be associated with limited health care resources, infrastructure, and access to quality breast cancer care, leading to lower survival rates. However, it is important to note that the Maajani et al study included data from both high-income and low-income countries, while breast cancer survival in Africa was underrepresented.

The 5-year pooled survival of breast cancer was higher (50.2% [95% CI, 46.2%-59.5%]) in a previous meta-analysis conducted in Africa.^70^ One possible explanation for this observed discrepancy could be the difference in the countries included in each study. Our study specifically focused on countries in SSA, which are generally characterized as the poorest countries in the region with the lowest economic growth of 3.4%.^71^ In contrast, the previous study^70^ pooled breast cancer survival data from all countries in Africa and indicated worst 5-year survival of breast cancer (35%) in the western region, followed by the eastern region (38%) and the southern region (48%), which are included in SSA. This interregional difference in survival rates could be attributed to variations in socioeconomic development and government health expenditure.^72^ However, it is important to note that this difference in breast cancer survival is not limited to interregional disparities but also extends to variations within countries, which can be explained by factors such as treatment access, social inequalities, and socioeconomic development at the country level.^62^

Notably, the southern region and non-SSA region exhibited higher socioeconomic development and government health expenditure compared with the eastern and western regions of Africa. This was accompanied by higher government health care spending per capita of US $221.6 in Tunisia and $214.8 in Algeria, compared with $93.00 in Ghana, $17.00 in Ethiopia, $59.00 in Tanzania, and $22.00 in Uganda.^73^ Therefore, it is crucial for governments in SSA regions to prioritize allocating more resources toward cancer prevention, early detection, and treatment that could help to improve access to quality health care services. Moreover, as many reports have indicated the advanced stage of diagnosis and poor breast cancer treatment adherence in SSA, efforts should focus on shifting to earlier stages at diagnosis and improving treatment adherence.^74^

The 5-year survival among men with breast cancer was lower (31%) than among women and/or populations of both men and women. This could be explained by the biological and genetic feature differences of male breast cancer.^75^ Male breast cancer cases are mostly hormone receptor–positive and are associated with an increased prevalence of *BRCA2* germline alterations, leading to more aggressive forms of breast cancer than in women.^75^ Moreover, male breast cancer typically presents at a later stage with larger tumor size, lymph node involvement, and distant metastases, resulting in poor prognosis and survival.^76^ Although the lower survival among men compared with women is in line with previous evidence, the 5-year survival among men in this study was much lower than the study from the US (79.1%).^77^ This could be attributed to the differences in health care access, quality, early detection, and treatment between SSA countries and the US.

Furthermore, the survival varied across each country’s HDI category. The survival rate was significantly lower in low-HDI countries compared with middle- and high-HDI countries. The evidence is consistent with that of previous studies showing a lower MIR among the high-HDI countries and a positive association between high HDI and breast cancer survival.^78,79^ The better survival rates among high-HDI countries could be attributed to the diagnosis of the disease in the earlier stages, the higher level of care, and better access to proper treatment. It was also reported that universal health coverage is positively correlated with HDI and is negatively correlated with the MIR. Furthermore, in low- and medium-HDI countries, most cancer care units are located in urban areas, which are often inaccessible to a significant proportion of the population residing in rural areas.^5^ Health literacy and the availability of diagnostic and treatment infrastructure such as radiological instruments and physicians are also other important barriers that affect survival in low-HDI countries.^5^ These results highlight the need for improving health care infrastructure, access, and awareness in countries with lower levels of development.

The subgroup analysis based on the study period showed a higher 3-year survival in studies conducted in recent years compared with those performed in previous decades. This is consistent with previous research indicating that breast cancer survival rates have increased over time.^80^ This increase is potentially attributable to advances in diagnostics, screening programs, and treatment guidelines over the past few decades.^69^

The subgroup analysis and meta-regression yielded an association between study quality and a 1-year survival rate. Good-quality studies exhibited better survival rates, which could be attributed to robust methods used in study design, data collection, and outcome ascertainment. However, the number of studies in each category of study quality was unbalanced. Particularly for 1-year survival, only 1 study was categorized as fair quality, which reported an exceptionally lower survival rate (0.29) compared with poor-quality (0.68) and good-quality (0.87) studies. Furthermore, we used a modified version of the Newcastle-Ottawa Scale to assess the study quality, which is prone to some level of reviewers’ subjectivity in evaluating certain items of the scale. Although we used a double risk of bias, it is possible that some degree of subjectivity remains. Therefore, the subgroup difference in study quality estimates may not be entirely reliable due to the unbalanced distribution of studies across each category and the inherent subjectivity in the assessment process. Further investigation of the study quality assessment and its potential impact on survival estimate is needed.

### Limitations

This study has some limitations. The findings may not represent all SSA countries. Some countries with many studies may be overrepresented, while those with few or no studies may not be well represented. The study only included individuals with confirmed breast cancer diagnoses, limiting generalizability to populations with limited access to screening and diagnostics. Last, we could not report stage-specific survival rates due to limited data.

## Conclusions

The survival rates of breast cancer in SSA are lower compared with global and other contexts. This systematic review and meta-analysis shows variation in survival rates across different subgroups, such as by country HDI and study period. Higher rates were observed in high-HDI countries and recent studies. The findings suggest that accessibility, health care quality, and affordability of services could be the underlying factors that influence survival of patients with breast cancer in SSA. To address this inequality, governments, clinicians, and stakeholders must work together to develop and implement tailored breast cancer screening and treatment programs that meet the specific needs of patients.

## Notes

Supplement 1. **eTable 1.** Literature Search Strategy **eTable 2.** Characteristics of the Included Articles **eFigure 1.** Risk of Bias of Included Studies by Study Period Based on the Newcastle-Ottawa Scale **eFigure 2.** Forest Plots Indicating the 2-Year and 4-Year Survival Rates of Patients With Breast Cancer in Sub-Saharan Africa **eFigure 3.** Forest Plot Indicating the 5-Year Survival Rates of Breast Cancer Among Male Patients in Sub-Saharan Africa **eFigure 4.** Forest Plots Indicating Subgroup Analysis by Human Development Index, Study Period and Study Quality **eFigure 5.** Funnel Plots Indicating Publication Bias Assessment by Study Year
eReferences

Supplement 2.
Data Sharing Statement

## References

[1] Azamjah N, Soltan-Zadeh Y, Zayeri F. Global trend of breast cancer mortality rate: a 25-year study. Asian Pac J Cancer Prev. 2019;20(7):2015-2020. doi:10.31557/APJCP.2019.20.7.2015 31350959 PMC6745227

[2] Huang J, Chan PS, Lok V, . Global incidence and mortality of breast cancer: a trend analysis. Aging (Albany NY). 2021;13(4):5748-5803. doi:10.18632/aging.202502 33592581 PMC7950292

[3] Xu S, Liu Y, Zhang T, . The global, regional, and national burden and trends of breast cancer from 1990 to 2019: results from the Global Burden of Disease Study 2019. Front Oncol. 2021;11:689562. doi:10.3389/fonc.2021.689562 34094989 PMC8176863

[4] Arnold M, Morgan E, Rumgay H, . Current and future burden of breast cancer: global statistics for 2020 and 2040. Breast. 2022;66:15-23. doi:10.1016/j.breast.2022.08.010 36084384 PMC9465273

[5] Sharma R. Breast cancer incidence, mortality and mortality-to-incidence ratio (MIR) are associated with human development, 1990-2016: evidence from Global Burden of Disease Study 2016. Breast Cancer. 2019;26(4):428-445. doi:10.1007/s12282-018-00941-4 30604398

[6] Azadnajafabad S, Saeedi Moghaddam S, Mohammadi E, . Patterns of better breast cancer care in countries with higher human development index and healthcare expenditure: insights from GLOBOCAN 2020. Front Public Health. 2023;11:1137286. doi:10.3389/fpubh.2023.1137286 37124828 PMC10130425

[7] World Health Organization. Breast cancer. March 13, 2024. Accessed July 24, 2023. https://www.who.int/news-room/fact-sheets/detail/breast-cancer

[8] DeSantis CE, Bray F, Ferlay J, Lortet-Tieulent J, Anderson BO, Jemal A. International variation in female breast cancer incidence and mortality rates. Cancer Epidemiol Biomarkers Prev. 2015;24(10):1495-1506. doi:10.1158/1055-9965.EPI-15-0535 26359465

[9] Sighoko D, Kamaté B, Traore C, . Breast cancer in pre-menopausal women in West Africa: analysis of temporal trends and evaluation of risk factors associated with reproductive life. Breast. 2013;22(5):828-835. doi:10.1016/j.breast.2013.02.011 23489760

[10] Verhoeven D, Allemani C, Kaufman C, Mansel R, Siesling S, Anderson B. Breast cancer: global quality care optimizing care delivery with existing financial and personnel resources. ESMO Open. 2020;4(suppl 2):e000861. doi:10.1136/esmoopen-2020-000861 32895233 PMC7478129

[11] Jemal A, Bray F, Forman D, . Cancer burden in Africa and opportunities for prevention. Cancer. 2012;118(18):4372-4384. doi:10.1002/cncr.27410 22252462

[12] Ziegenhorn HV, Frie KG, Ekanem IO, . Breast cancer pathology services in sub-Saharan Africa: a survey within population-based cancer registries. BMC Health Serv Res. 2020;20(1):912. doi:10.1186/s12913-020-05752-y 33008380 PMC7531092

[13] Coughlin SS. Social determinants of breast cancer risk, stage, and survival. Breast Cancer Res Treat. 2019;177(3):537-548. doi:10.1007/s10549-019-05340-7 31270761

[14] Gebremariam A, Addissie A, Worku A, . Association of delay in breast cancer diagnosis with survival in Addis Ababa, Ethiopia: a prospective cohort study. JCO Glob Oncol. 2023;9:e2300148. doi:10.1200/GO.23.00148 37992269 PMC10681531

[15] World Health Organization. The Global breast cancer initiative. Accessed August 20, 2023. https://www.who.int/initiatives/global-breast-cancer-initiative

[16] Ouzzani M, Hammady H, Fedorowicz Z, Elmagarmid A. Rayyan-a web and mobile app for systematic reviews. Syst Rev. 2016;5(1):210. doi:10.1186/s13643-016-0384-4 27919275 PMC5139140

[17] Cramond F, O’Mara-Eves A, Doran-Constant L, Rice AS, Macleod M, Thomas J. The development and evaluation of an online application to assist in the extraction of data from graphs for use in systematic reviews. Wellcome Open Res. 2019;3:157. doi:10.12688/wellcomeopenres.14738.3 30809592 PMC6372928

[18] Egger M, Davey Smith G, Schneider M, Minder C. Bias in meta-analysis detected by a simple, graphical test. BMJ. 1997;315(7109):629-634. doi:10.1136/bmj.315.7109.629 9310563 PMC2127453

[19] The United Nation’s Development Program. Human development report 2021/2022: uncertain times, unsettled lives, shaping our future in a transforming world. Accessed August 20, 2023. https://www.undp.org/sites/g/files/zskgke326/files/2023-01/Human%20Development%20Report%2020212022%20Overview%20-%20English.pdf

[20] Olayide A, Samuel O, Ganiyu R, . How effective is the treatment of locally advanced and metastatic breast cancer in developing centres? a retrospective review. Ethiop J Health Sci. 2015;25(4):337-344. doi:10.4314/ejhs.v25i4.7 26949298 PMC4762972

[21] Ahmed A, Ukwenya Y, Abdullahi A, Muhammad I. Management and outcomes of male breast cancer in Zaria, Nigeria. Int J Breast Cancer. 2012;2012:845143. doi:10.1155/2012/845143 22991670 PMC3443591

[22] Ajayi DOS, Osegbe DN, Ademiluyi SA. Carcinoma of the male breast in West Africans and a review of world literature. Cancer. 1982;50(8):1664-1667. doi:10.1002/1097-0142(19821015)50:8<1664::AID-CNCR2820500834>3.0.CO;2-N 6288222

[23] Ali-Gombe M, Mustapha MI, Folasire A, Ntekim A, Campbell OB. Pattern of survival of breast cancer patients in a tertiary hospital in South West Nigeria. ecancermedicalscience. 2021;15:1192. doi:10.3332/ecancer.2021.1192 PMC8043689 33889201

[24] Arowolo OA, Akinkuolie AA, Lawal OO, Alatise OI, Salako AA, Adisa AO. The impact of neoadjuvant chemotherapy on patients with locally advanced breast cancer in a Nigerian semiurban teaching hospital: a single-center descriptive study. World J Surg. 2010;34(8):1771-1778. doi:10.1007/s00268-010-0617-y 20467742

[25] Ayandipo OO, Afuwape OO, Adepoju OJ, Ajiboye JA, Ogundiran TO. Stage-specific five-year survival outcomes in women treated for early stage breast cancer in Ibadan, Nigeria. Niger J Med. 2020;29(1):152-157. doi:10.4103/1115-2613.284879

[26] Ayandipo OO, Ogun GO, Adepoju OJ, . Impact of axillary node-positivity and surgical resection margins on survival of women treated for breast cancer in Ibadan, Nigeria. Ecancermedicalscience. 2020;14:1084. doi:10.3332/ecancer.2020.1084 32863878 PMC7434507

[27] Ayoade B, Agboola A, Olatunji A, Tade A, Salami B, Adekoya A. Clinical characteristics and survival outcome of breast cancer in Southwest Nigerian women. Afr J Cancer. 2014;2(6):79-84. doi:10.1007/s12558-014-0311-8

[28] Ntekim AI, Folasire AM, Ali-Gombe M. Survival pattern of rare histological types of breast cancer in a Nigerian institution. Pan Afr Med J. 2019;34(1):114. doi:10.11604/pamj.2019.34.114.16925 31934255 PMC6945379

[29] Okobia MN, Osime U. Clinicopathological study of carcinoma of the breast in Benin City. Afr J Reprod Health. 2001;5(2):56-62. doi:10.2307/3583430 12471913

[30] Olasehinde O, Alatise O, Omisore A, . Contemporary management of breast cancer in Nigeria: insights from an institutional database. Int J Cancer. 2021;148(12):2906-2914. doi:10.1002/ijc.33484 33506499 PMC8394611

[31] Otu AA, Ekanem IO, Khalil MI, Ekpo MD, Attah EB. Characterization of breast cancer subgroups in an African population. Br J Surg. 1989;76(2):182-184. doi:10.1002/bjs.1800760225 2702455

[32] Wuraola FO, Olasehinde O, Di Bernardo M, . Breast cancer in elderly patients: a clinicopathological review of a Nigerian database. Ecancermedicalscience. 2022;16:1484. doi:10.3332/ecancer.2022.1484 36819793 PMC9934965

[33] Eber-Schulz P, Tariku W, Reibold C, . Survival of breast cancer patients in rural Ethiopia. Breast Cancer Res Treat. 2018;170(1):111-118. doi:10.1007/s10549-018-4724-z 29479644

[34] Shibabaw W, Mulugeta T, Abera H, Asmare Y, Yirga T. Survival status and predictors of mortality among breast cancer patients at Black Lion specialized hospital, adult oncology unit, Addis Ababa, Ethiopia, 2018: a retrospective follow-up study with survival analysis. bioRxiv. Preprint posted online July 21, 2019. doi:10.1101/636431

[35] Tessema Ersumo M, Girmaye Tamrat M, Bogale Solomon M, Tariku Gero M. Breast cancer in a private medical services center: a 10-year experience. Ethiopian Med J. 2018;56(3):211-218.

[36] Tiruneh M, Tesfaw A, Tesfa D. Survival and predictors of mortality among breast cancer patients in Northwest Ethiopia: a retrospective cohort study. Cancer Manag Res. 2021;13:9225-9234. doi:10.2147/CMAR.S339988 34938122 PMC8687444

[37] Basro S, Apffelstaedt JP. Breast cancer in young women in a limited-resource environment. World J Surg. 2010;34(7):1427-1433. doi:10.1007/s00268-009-0299-5 19997919

[38] Cubasch H, Dickens C, Joff M, . Breast cancer survival in Soweto, Johannesburg, South Africa: a receptor-defined cohort of women diagnosed from 2009 to 11. Cancer Epidemiol. 2018;52:120-127. doi:10.1016/j.canep.2017.12.007 29306221 PMC6127863

[39] Du Plessis M, Apffelstaedt JP. Treatment outcomes of breast carcinoma in a resource-limited environment surgery. S Afr J Surg. 2015;53(2):43-47. doi:10.7196/sajsnew.7842

[40] Parag Y, Buccimazza I. How long are elderly patients followed up with mammography after the diagnosis of breast cancer? a single-centre experience in a developing country. S Afr Med J. 2016;106(7):721-723. doi:10.7196/SAMJ.2016.v106i7.10405 27384369

[41] Elhaj AM, Abdalsalam A, Abuidris A, Eltayeb A. Overall survival of females with breast cancer in the National Cancer Institute, University of Gezira, Sudan. Sudan Medical Monitor. 2015;10(1):1-6. doi:10.4103/1858-5000.157499

[42] Abdalla Elhassan SI. The five-year survival rate of breast cancer at Radiation and Isotopes Centre Khartoum, Sudan. Heliyon. 2020;6(8):e04615. doi:10.1016/j.heliyon.2020.e04615 32904288 PMC7452576

[43] Muddather HF, Elhassan MMA, Faggad A. Survival outcomes of breast cancer in Sudanese women: a hospital-based study. JCO Glob Oncol. 2021;7(1):324-332. doi:10.1200/GO.20.00538 33617296 PMC8081542

[44] Baako B, Badoe E. Treatment of breast cancer in Accra: 5-year survival. Ghana Med J. 2001;35(2):90-93.

[45] Mensah AC. Survival outcomes of breast cancer in Ghana: An analysis of clinicopathological features. OAlib. 2016;3(1):1-11. doi:10.4236/oalib.1102145

[46] Ssentongo P, Oh JS, Amponsah-Manu F, . Breast cancer survival in eastern region of Ghana. Front Public Health. 2022;10:880789. doi:10.3389/fpubh.2022.880789 35719670 PMC9201058

[47] Gakwaya A, Kigula-Mugambe JB, Kavuma A, . Cancer of the breast: 5-year survival in a tertiary hospital in Uganda. Br J Cancer. 2008;99(1):63-67. doi:10.1038/sj.bjc.6604435 18577991 PMC2453032

[48] Galukande M, Wabinga H, Mirembe F. Breast cancer survival experiences at a tertiary hospital in sub-Saharan Africa: a cohort study. World J Surg Oncol. 2015;13(1):220. doi:10.1186/s12957-015-0632-4 26187151 PMC4506617

[49] Gondos A, Brenner H, Wabinga H, Parkin DM. Cancer survival in Kampala, Uganda. Br J Cancer. 2005;92(9):1808-1812. doi:10.1038/sj.bjc.6602540 15827554 PMC2362045

[50] Somé OR, Bague AH, Konkobo D, . Breast cancer in Bobo-Dioulasso, Burkina Faso: management outcomes. Oncologie. 2022;24(2):173-184. doi:10.32604/oncologie.2022.021250

[51] Zongo N, Ouédraogo S, Bado C, Kaboré A, Dem A. Survival of patients operated on for breast cancer in Ouagadougou/Burkina Faso. Eur J Surg Oncol. 2022;48(12):2378-2384. doi:10.1016/j.ejso.2022.07.001 35871031

[52] Zongo N, Ouédraogo S, Korsaga-Somé N, . Male breast cancer: diagnosis stages, treatment and survival in a country with limited resources (Burkina Faso). World J Surg Oncol. 2018;16(1):4. doi:10.1186/s12957-017-1297-y 29325566 PMC5765600

[53] Degu A, Terefe EM, Some ES, Tegegne GT. Treatment outcomes and its associated factors among adult patients with selected solid malignancies at Kenyatta National Hospital: a hospital-based prospective cohort study. Cancer Manag Res. 2022;14:1525-1540. doi:10.2147/CMAR.S361485 35498512 PMC9042075

[54] Wambua MD, Degu A, Tegegne GT. Treatment outcomes and its associated factors among breast cancer patients at Kitui Referral Hospital. SAGE Open Med. 2022;10:20503121211067857. doi:10.1177/20503121211067857 35024144 PMC8744162

[55] Msyamboza KP, Manda G, Tembo B, . Cancer survival in Malawi: a retrospective cohort study. Pan Afr Med J. 2014;19(1):234. doi:10.11604/pamj.2014.19.234.4675 25838862 PMC4377240

[56] Youngblood VM, Nyirenda R, Nyasosela R, . Outcomes and prognostic factors for women with breast cancer in Malawi. Cancer Causes Control. 2020;31(4):393-402. doi:10.1007/s10552-020-01282-4 32124187 PMC7115156

[57] Ngowa JDK, Kasia JM, Yomi J, . Breast cancer survival in Cameroon: analysis of a cohort of 404 patients at the Yaoundé General Hospital. Adv Breast Cancer Res. 2015;4(2):44. doi:10.4236/abcr.2015.42005

[58] Zingue S, Atenguena EO, Zingue LL, . Epidemiological and clinical profile, and survival of patients followed for breast cancer between 2010 and 2015 at the Yaounde General Hospital, Cameroon. Pan Afr Med J. 2021;39(1):182. doi:10.11604/pamj.2021.39.182.26866 34466203 PMC8378266

[59] Guèye M, Guèye S, Mbaye M, . Clinical features and prognosis of triple negative breast cancer at the senology unit of Aristide-Le-Dantec Teaching Hospital. Afr J Cancer. 2013;5(1):42-47. doi:10.1007/s12558-013-0252-2

[60] Gueye M, Kane Gueye SM, Ndiaye Gueye MD, . Breast cancer in women younger than 35 years: features and outcomes in the breast unit at Aristide le Dantec Teaching Hospital, Dakar. Article in French. Med Sante Trop. 2016;26(4):377-381. doi:10.1684/mst.2016.0637 28073726

[61] Joko-Fru WY, Miranda-Filho A, Soerjomataram I, . Breast cancer survival in sub-Saharan Africa by age, stage at diagnosis and human development index: a population-based registry study. Int J Cancer. 2020;146(5):1208-1218. doi:10.1002/ijc.32406 31087650 PMC7079125

[62] McCormack V, McKenzie F, Foerster M, . Breast cancer survival and survival gap apportionment in sub-Saharan Africa (ABC-DO): a prospective cohort study. Lancet Glob Health. 2020;8(9):e1203-e1212. doi:10.1016/S2214-109X(20)30261-8 32827482 PMC7450275

[63] Bah E, Sam O, Whittle H, Ramanakumar A, Sankaranarayanan R. Cancer survival in the Gambia, 1993-1997. IARC Sci Publ. 2011;(162):97-100. 21675410

[64] Chokunonga E, Borok MZ, Chirenje ZM, Nyabakau AM, Parkin DM. Cancer survival in Harare, Zimbabwe, 1993-1997. IARC Sci Publ. 2011;162(162):249-255. 21675430

[65] Mabula JB, Mchembe MD, Chalya PL, . Stage at diagnosis, clinicopathological and treatment patterns of breast cancer at Bugando Medical Centre in north-western Tanzania. Tanzan J Health Res. 2012;14(4):269-279. doi:10.4314/thrb.v14i4.6 26591725

[66] Brandão M, Guisseve A, Bata G, . Breast cancer subtypes: implications for the treatment and survival of patients in Africa-a prospective cohort study from Mozambique. ESMO Open. 2020;5(5):e000829. doi:10.1136/esmoopen-2020-000829 33020218 PMC7537337

[67] N’koua-M’bon JB, Bambara AT, Moukassa D, Gombé-Mbalawa C. Clinical and outcome characteristics of inflammatory breast cancers in Brazzaville. Article in French. Bull Cancer. 2013;100(2):147-153. doi:10.1684/bdc.2013.1700 23392567

[68] Traore B, Keita M, Toure A, Camara I, Barry A, Koulibaly M. Impact of surgery associated with radiotherapy on the prognosis of breast cancer—Guinea Breast Cancer Cohort Study. Cancer Rep (Hoboken). 2022;5(9):e1554. doi:10.1002/cnr2.1554 34549551 PMC9458488

[69] Maajani K, Jalali A, Alipour S, Khodadost M, Tohidinik HR, Yazdani K. The global and regional survival rate of women with breast cancer: a systematic review and meta-analysis. Clin Breast Cancer. 2019;19(3):165-177. doi:10.1016/j.clbc.2019.01.006 30952546

[70] Ssentongo P, Lewcun JA, Candela X, . Regional, racial, gender, and tumor biology disparities in breast cancer survival rates in Africa: a systematic review and meta-analysis. PLoS One. 2019;14(11):e0225039. doi:10.1371/journal.pone.0225039 31751359 PMC6872165

[71] International Monetary Fund. Regional economic outlook: sub-Saharan Africa. 2022. Accessed October 13, 2023. https://www.imf.org/en/Publications/REO/SSA

[72] Chireshe J, Ocran MK. Financial development and health care expenditure in sub Saharan Africa Countries. Cogent Econ Finance. 2020;8(1):1771878. doi:10.1080/23322039.2020.1771878

[73] The World Bank. Current health expenditure per capita (current US$)—Middle East & North Africa. April 7, 2023. Accessed August 8, 2023. https://data.worldbank.org/indicator/SH.XPD.CHEX.PC.CD?locations=ZQ

[74] Pace LE, Shulman LN. Breast cancer in sub-Saharan Africa: challenges and opportunities to reduce mortality. Oncologist. 2016;21(6):739-744. doi:10.1634/theoncologist.2015-0429 27091419 PMC4912363

[75] Gucalp A, Traina TA, Eisner JR, . Male breast cancer: a disease distinct from female breast cancer. Breast Cancer Res Treat. 2019;173(1):37-48. doi:10.1007/s10549-018-4921-9 30267249 PMC7513797

[76] Anderson WF, Jatoi I, Tse J, Rosenberg PS. Male breast cancer: a population-based comparison with female breast cancer. J Clin Oncol. 2010;28(2):232-239. doi:10.1200/JCO.2009.23.8162 19996029 PMC2815713

[77] Yadav S, Karam D, Bin Riaz I, . Male breast cancer in the United States: treatment patterns and prognostic factors in the 21st century. Cancer. 2020;126(1):26-36. doi:10.1002/cncr.32472 31588557 PMC7668385

[78] Hu K, Lou L, Tian W, Pan T, Ye J, Zhang S. The outcome of breast cancer is associated with national human development index and health system attainment. PLoS One. 2016;11(7):e0158951. doi:10.1371/journal.pone.0158951 27391077 PMC4938431

[79] Zolghadr Z, Salehi M, Dehnad A, Zayeri F. A study of relationship between breast cancer mortality rate and human development index: global trend analysis from 1990 to 2017. Int J Cancer Manag. 2020;13(8):e101813. doi:10.5812/ijcm.101813

[80] Giordano SH, Buzdar AU, Smith TL, Kau SW, Yang Y, Hortobagyi GN. Is breast cancer survival improving? Cancer. 2004;100(1):44-52. doi:10.1002/cncr.11859 14692023

