# Supplementary material for: Survival Patterns Among Patients With Breast Cancer in Sub-Saharan Africa: A Systematic Review and Meta-Analysis
Source: JAMA Netw Open. 2024 May 14;7(5):e2410260. doi: 10.1001/jamanetworkopen.2024.10260 (PMC11094564; doi:10.1001/jamanetworkopen.2024.10260)
Supplement: Supplement 1. — eTable 1. Literature Search Strategy eTable 2. Characteristics of the Included Articles eFigure 1. Risk of Bias of Included Studies by Study Period Based on the Newcastle-Ottawa Scale eFigure 2. Forest Plots Indicating the 2-Year and 4-Year Survival Rates of Patients With Breast Cancer in Sub-Saharan Africa eFigure 3. Forest Plot Indicating the 5-Year Survival Rates of Breast Cancer Among Male Patients in Sub-Saharan Africa eFigure 4. Forest Plots Indicating Subgroup Analysis by Human Development Index, Study Period and Study Quality eFigure 5. Funnel Plots Indicating Publication Bias Assessment by Study Year eReferences [file jamanetwopen-e2410260-s001.pdf]

Supplementary Online Content

Limenih MA, Mekonnen EG, Birhanu F, et al. Survival patterns among patients with breast cancer in sub-Saharan Africa: a systematic review and meta-analysis. *JAMA Netw Open*. 2024;7(5):e2410260. doi:10.1001/jamanetworkopen.2024.10260

eTable 1. Literature Search Strategy

eTable 2. Characteristics of the Included Articles

eFigure 1. Risk of Bias of Included Studies by Study Period Based on the Newcastle-Ottawa Scale

eFigure 2. Forest Plots Indicating the 2-Year and 4-Year Survival Rates of Patients With Breast Cancer in Sub-Saharan Africa

eFigure 3. Forest Plot Indicating the 5-Year Survival Rates of Breast Cancer Among Male Patients in Sub-Saharan Africa

eFigure 4. Forest Plots Indicating Subgroup Analysis by Human Development Index, Study Period and Study Quality

eFigure 5. Funnel Plots Indicating Publication Bias Assessment by Study Year

eReferences

This supplementary material has been provided by the authors to give readers additional information about their work.

© 2024 Limenih MA et al. *JAMA Network Open*.

eTable 1. Literature Search Strategy

Box S1: Search strategy in PubMed/ Medline

#1	((((((breast)) OR (breast [MeSH Terms])) OR ((lobular))) OR ((ductal))) OR ((mammary))) OR ((adipose tissue))
#2	(((((((((cancer [MeSH Terms]) OR (cancer*)) OR (neoplasm*)) OR (carcinoma*)) OR (sarcoma*)) OR (tumor*)) OR (tumour*)) OR (malignan*)) OR (Neoplasia)) OR (Carcino*)
#3	((((((((survival) OR (survival analysis [MeSH Terms])) OR ((survival analysis))) OR ((Survival rate*))) OR (mortality)) OR (prognosis)) OR (prognostic)) OR ((Proportional Hazards Models))) OR (frailty*)
#4	(((Africa) OR ((sub-Sahara* Africa))) OR ((south Sahara desert))) OR (Sahel) OR (Angola OR Benin OR Botswana OR (Burkina Faso) OR Burundi OR Cameroun OR Cameron OR (Cape Verde) OR Chad OR Comoros OR Congo OR (Cote d'Ivoire) OR (Ivory Coast) OR (Democratic Republic Congo) OR (Equatorial Guinea) OR Eritrea OR Ethiopia OR Gabon OR Gambia OR Ghana OR Guinea OR (Guinea Bissau) OR Kenya OR Lesotho OR Liberia OR Madagascar OR Malawi OR Mali OR Mauritania OR Mauritius OR Mozambique OR Namibia OR Niger OR Nigeria OR Rwanda OR (Sao Tome) OR Senegal OR Seychelles OR (Sierra Leone) OR Somalia OR (South Sudan) OR (South Africa) OR Sudan OR Swaziland OR Togo OR Uganda OR Tanzania OR Zambia OR Zimbabwe)
#4	#1 AND #2 AND #3 AND #4
#5	Filters: year of publication: from January 1st, 1943, to December 31st, 2022; Population: Humans

© 2024 Limenih MA et al. *JAMA Network Open*.

Box S2: Search strategy in Embase

#1	(Breast) OR ductal OR lobular OR mammary OR 'adipose tissue'
#2	(cancer* OR neoplasm* OR carcinoma* OR sarcoma* OR tumor* OR tumour* OR malignan* OR Neoplasia OR Carcino*)
#3	(Survival OR 'Survival analysis' OR 'Survival rate*' OR mortality OR prognosis OR prognostic OR 'Proportional Hazards Models' OR frailty*)
#4	(Africa OR 'sub-Saharan Africa' OR Sub-Saharan OR Angola OR Benin OR Botswana OR 'Burkina Faso' OR Burundi OR Cameroun OR 'Cape Verde' OR Chad OR Comoros OR Congo OR 'Cote dIvoire Ivory Coast' OR 'Democratic Republic of Congo' OR 'Equatorial Guinea' OR Eritrea OR Ethiopia OR Gabon OR Gambia OR Ghana OR Guinea OR Guinea-Bissau OR Kenya OR Lesotho OR Liberia OR Madagascar OR Malawi OR Mali OR Mauritania OR Mauritius OR Mozambique OR Namibia OR Niger OR Nigeria OR Rwanda OR 'Sao Tome' OR Senegal OR Seychelles OR 'Sierra Leone' OR Somalia OR 'South Sudan' OR 'South Africa' OR Sudan OR Swaziland OR Togo OR Uganda OR Tanzania OR Zambia OR Zimbabwe)
#4	#1 AND #2 AND #3 AND #4
#5	Filters: year of publication: from January 1st, 1943, to December 31st, 2022; Population: Humans

© 2024 Limenih MA et al. *JAMA Network Open*.

Box S3: Search strategy in Web-of science

#1	(((((TS= (Breast)) OR TS=(ductal)) OR TS=(lobular)) OR TS=(mammary)) OR TS= (adipose tissue))
#2	(((((((((((((((((TS=(cancer*)) OR TS=(neoplasm*)) OR TS=(carcinoma*)) OR TS=(sarcoma*)) OR TS=(tumor*)) OR TS=(tumour*)) OR TS=(malignan*)) OR TS=(Neoplasia)) OR TS=(Carcino*)) AND TS=(Survival)) OR TS=(Survival analysis)) OR TS=(Survival rate)) OR TS=(mortality)) OR TS=(prognosis)) OR TS=(prognostic)) OR TS=(Proportional Hazards Models)) OR TS=(frailty*))
#3	(((TS=(Africa)) OR TS=(sub-Saharan Africa)) OR TS=(Sub-Saharan)) OR TS=(Angola)) OR TS=(Benin)) OR TS=(Botswana)) OR TS=(Burkina Faso)) OR TS=(Burundi)) OR TS=(Cameroun)) OR TS=(Cameron)) OR TS=(Cape Verde)) OR TS=(Chad)) OR TS=(Comoros)) OR TS=(Congo)) OR TS=(Cote d'Ivoire Ivory Coast)) OR TS=(Democratic Republic of Congo)) OR TS=(Equatorial Guinea)) OR TS=(Eritrea)) OR TS=(Ethiopia)) OR TS=(Gabon)) OR TS=(Gambia)) OR TS=(Ghana)) OR TS=(Guinea)) OR TS=(Guinea-Bissau)) OR TS=(Kenya)) OR TS=(Lesotho)) OR TS=(Liberia)) OR TS=(Madagascar)) OR TS=(Malawi)) OR TS=(Mali)) OR TS=(Mauritania)) OR TS=(Mauritius)) OR TS=(Mozambique)) OR TS=(Namibia)) OR TS=(Niger)) OR TS=(Nigeria)) OR TS=(Rwanda)) OR TS=(Sao Tome)) OR TS=(Senegal)) OR TS=(Seychelles)) OR TS=(Sierra Leone)) OR TS=(Somalia)) OR TS=(South Sudan)) OR TS=(South Africa)) OR TS=(Sudan)) OR TS=(Swaziland)) OR TS=(Togo)) OR TS=(Uganda)) OR TS=(Tanzania)) OR TS=(Zambia)) OR TS=(Zimbabwe)
#3	#1 AND #2 AND #3 AND #4
#4	Filters: year of publication: from January 1st, 1943, to December 31st, 2022; Population: Humans

© 2024 Limenih MA et al. *JAMA Network Open*.

Box S4: Search strategy in Scopus

#1	(Breast) OR (ductal) OR (lobular) OR (mammary) OR (adipose AND tissue)
#2	cancer* OR neoplasm* OR carcinoma* OR sarcoma* OR tumor* OR tumour* OR malignan* OR Neoplasia OR Carcino*
#3	Survival OR "Survival analysis" OR "Survival rate*" OR mortality OR prognosis OR prognostic OR "Proportional Hazards Models" OR frailty*
#4	Africa OR "sub-Saharan Africa" OR Sub-Saharan OR Angola OR Benin OR Botswana OR "Burkina Faso" OR Burundi OR Cameroun OR "Cape Verde" OR Chad OR Comoros OR Congo OR "Cote dIvoire Ivory Coast" OR "Democratic Republic of Congo" OR "Equatorial Guinea" OR Eritrea OR Ethiopia OR Gabon OR Gambia OR Ghana OR Guinea OR Guinea-Bissau OR Kenya OR Lesotho OR Liberia OR Madagascar OR Malawi OR Mali OR Mauritania OR Mauritius OR Mozambique OR Namibia OR Niger OR Nigeria OR Rwanda OR "Sao Tome" OR Senegal OR Seychelles OR "Sierra Leone" OR Somalia OR "South Sudan" OR "South Africa" OR Sudan OR Swaziland OR Togo OR Uganda OR Tanzania OR Zambia OR Zimbabwe
#4	#1 AND #2 AND #3 AND #4
#5	Filters: year of publication: from January 1st, 1943, to December 31st, 2022; Population: Humans

© 2024 Limenih MA et al. *JAMA Network Open*.

Box S5: Search strategy in Google Scholar

#1	“Breast cancer” AND “survival” AND Africa OR “sub-Sahara Africa”
#2	"Breast cancer survival" AND Africa OR “sub-Sahara Africa”
#3	**(**Breast) AND (cancer) AND (Survival) AND ((sub-Saharan Africa) OR Angola OR Benin OR Botswana OR (Burkina Faso) OR Burundi OR Cameroun OR Cameron OR (Cape Verde) OR Chad OR Comoros OR Congo OR (Cote d'Ivoire) OR (Ivory Coast) OR (Democratic Republic of Congo) OR (Equatorial Guinea) OR Eritrea OR Ethiopia OR Gabon OR Gambia OR Ghana OR Guinea OR (Guinea-Bissau) OR Kenya OR Lesotho OR Liberia OR Madagascar OR Malawi OR Mali OR Mauritania OR Mauritius OR Mozambique OR Namibia OR Niger OR Nigeria OR Rwanda OR (Sao Tome) OR Senegal OR Seychelles OR (Sierra Leone) OR Somalia OR (South Sudan) OR (South Africa) OR Sudan OR Swaziland OR Togo OR Uganda OR Tanzania OR Zambia OR Zimbabwe)
#4	Filters: year of publication: from January 1st, 1943, to December 31st, 2022; Population: Humans

© 2024 Limenih MA et al. *JAMA Network Open*.

eTable 2. Characteristics of the Included Articles

**Author, Year, Country**	**Sample size**	**Sex**	**Time span**	**FU duration (years)**	**Age (mean/ median)**	**Death ascertainment**	**Median FU month**	**Median survival (month)(95%CI)**	**Survival rates (95%CI)**					
									1-year	2-year	3-year	4-year	5-year	10-year
**Elhassan,[19] 2020 (Elhassan, 2020), Sudan**	305	Both	2013-NR	5	NR	Verbal autopsy and medical record	NR	NR	NR	NR	NR	NR	79.0 (74.0,84.0)	NR
**Ajayi,[3] 1982 (Ajayi et al., 1982), Nigeria**	30	Male	1971-1980	9	56.3	NR	NR	NR	NR	NR	NR	NR	22.0 (7.1-36.9)	NR
**Ahmed,[2] 2012 (Ahmed et al., 2012), Nigeria**	57	Male	2001-2010	9	59 (2.3)	NR	NR	NR	NR	NR	NR	NR	23.0 (12.0-35.0)	NR
**Arowolo,[5] 2010 (Arowolo et al., 2010), Nigeria**	62	Both	1982-2005	5	49.1 (12.7)	NR	9	NR	66.0(54.0-0.79)	42.0(29.0-59.0)	NR	NR	12.0 (4.0-19.0)	NR
**Ayandipo,[6] 2020 (Ayandipo et al., 2020b), Nigeria**	225	Female	2009-2014	5	48.6 (11.8)	Phone calls and medical record	NR	NR	NR	NR	NR	NR	65.3 (56.8-72.0)	NR
**Ayandipo[7] 2020 (Ayandipo et al., 2020a), Nigeria**	63	Female	2013-2018	5	43 (10)	NR	NR	47	NR	NR	NR	NR	78.0 (77.7-77.9)	NR
**Ayoade,[8] 2014 (Ayoade et al., 2014), Nigeria**	139	Female	2004-2008	5	48.1 (11.7)	NR	NR	33	86.0(80.2-91.8)	82.0(76.4-87.6)	72.0(64.9 -79.1)	48.0(40.6-55.4)	NR	NR
**Baako,[9] 2001 (Baako and Badoe, 2001), Ghana**	145	Both	1990-1998	8	47	Medical record	NR	NR	NR	63.9(55.1-72.7)	NR	NR	25.3 (18.4-32.2)	NR
**Bah,[10] 2011 (Bah et al., 2011), Gambia**	61	Both	1993_1997	5	NR	Death certificate, medical record, and house visit	4.6	NR	29.0(19.0-41.0)	NR	NR	NR	9.3 (2.0-16.0)	NR
**Basro,[11] 2010 (Basro and Apffelstaedt, 2010), South Africa**	141	Female	2000-2008	7.5	NR	NR	18	31	NR	46.5(36.9-55.1)	NR	NR	20.0 (13.2-26.8)	NR
**Chokunonga,[13] 2011 (Chokunonga et al., 2011), Zimbabwe**	258	Both	1993-1997	NR	NR	Death Certificate	39.3	NR	81.1(75.5-86.7)		62.7(56.5 -68.9)	NR	51.2 (44.4-58.0)	
**Cubasch,[14] 2018 (Cubasch et al., 2018), SA**	604	Female	2009-2014	5	54.4 (14.2)	Medical record	25	NR	92.0(90.0-94.0)	83.0(80.2-85.8)	72.0(68.5 -75.5)	67.0(63.2-70.8)	63.0 (59.2-66.8)	NR
**Degu,[15] 2022 (Degu et al., 2022), Kenya**	99	Female	2020-2021	1	49.2 (12.1)	NR	NR	NR	95.0(92.9-97.1)	NR	NR	NR	NR	NR
**Elhaj,[18] 2015 (Elhaj et al., 2015), Sudan**	64	Female	2001-2006	5	NR	Medical record and verbal autopsy	10.5	40	50.0(37.5-62.5)	NR	NR	NR	38.8 (23.6-52.4)	NR
**Ersumo,[41] 2018 (Tessema Ersumo et al., 2018), Ethiopian**	370	Both	2005-2015	10	45.9 (11.9)	Medical record	NR	NR	NR	NR	NR	NR	69.5 (64 .3-74.4)	NR
**Gakwaya,[20] 2008 (Gakwaya et al., 2008), Uganda**	297	Both	199-2000	5	47	NR	NR	NR	77.0(72.1-81.9)	NR	NR	NR	56.0 (50.2-61.8)	NR

© 2024 Limenih MA et al. *JAMA Network Open*.

**Galukande,[21] 2015 (Galukande et al., 2015), Uganda**	262	Female	2004-2010, 2007-2012	2 and 5	45.5	Death certificate and medical record	NR	NR	NR	NR	NR	NR	51.8 (44.6-59.0)	NR
**Ali-Gombe,[4] 2021 (Ali-Gombe et al., 2021), Nigeria**	378	Both	2008-2013	5	47.6 (11.2)	Medical record and verbal autopsy	NR	41	84.2(80.5-87.9)	56.1(50.8-61.4)	54.8(49.5 -60.1)	45.3(38.6-48.4)	37.6 (32.5-42.7)	NR
**Gondos,[22] 2005 (Gondos et al., 2005), Uganda**	174	Both	2001-2005	4	45	Verbal autopsy	NR	NR	NR	NR	NR	NR	38.4 (31.6-45.2)	NR
**Gueye,[23] 2013 (Guèye et al., 2013), Senegal**	22	Female	2009-2012	3	45.4	NR	NR	25	NR	NR	NR	NR	41.0 (17.0-68)	NR
**Gueye,[24] 2016 (Gueye et al., 2016), Senegal**	62	Female	2007-2015	5	30 (Range;18-34)	Medical record	NR	39.7	81.5(71.4-89.6)	65.4(53.5-77.3)	55.9(44.2 -67.6)	49.7(37.8-61.6)	44.8 (32.9-56.7)	NR
**Joko-Fru,[25] 2020 (Joko-Fru et al., 2020), Multicounty¥**	2311	Female	2008-2017	10	52.7	Death certificate, medical record, and house visit	NR	55.2	84.1(82.5-85.6)	NR	61.4(59.4 -63.4)	NR	52.3 (49.9-54.6)	NR
**Mabula,[26] 2012 (Mabula et al., 2012), Tanzania**	384	Both	2002-2011	10	NR	NR	12	NR	24.0(19.1-28.9)	NR	NR	NR	21.8 (16.8-26.8)	NR
**Mariana, 2020 (Brandão et al., 2020), Mozambique**	210	Female	2017-2019	3	48	Medical record and verbal autopsy	38.3	NR	NR	NR	52.5(44.8 -60.2)	NR	NR	NR
**McCormack,[27] 2020 (McCormack et al., 2020), Multicounty***	2156	Female	2014-2017	3	52(14)	MEDICAL RECORD, Verbal autopsy	33.6	11.9	79.0(77.0-81.0)	NR	50.0(48.2 -51.8)	NR	NR	NR
**Mensah,[28] 2016 (Mensah, 2016), Ghana**	1022	Female	2002-2011	9	43.5(12.5)	Medical record, verbal autopsy	NR	59.2	81.5(78.9-84.0)	65.4(53.5-77.3)	55.9(44.2 -67.6)	49.7(37.4-61.4)	47.9 (36.1-59.7)	NR
**Msyamboza,[29] 2014 (Msyamboza et al., 2014), Malawi**	21	Female	2006-2013	7	NR	NR	NR	5.6(4.9-6.3)	9.5(5.0-24.1)	NR	NR	NR	NR	NR
**Muddather,[30] 2021 (Muddather et al., 2021), Sudan**	225	Female	2012-2018	6	45	Medical record, verbal autopsy	59.8	66.7	87.6(82.7-91.6)	73.2(66.3-80.1)	67.8(60.7 -74.9)	61.6(54.4-68.8)	58.2 (50.8-66.2)	NR
**Ngowa,[32] 2015 (Ngowa et al., 2015), Cameroon**	221	Female	1995-2010	10	47.5(12.4)	Medical record, verbal autopsy	NR	24(22.8-36)	NR	NR	NR	NR	30.0 (24.6-35.4)	13.2(9.3-17.1)
**N'Koua-M'Bon,[31] 2013 (N'Koua-M'bon et al., 2013), Congo**	129	Female	2007-2010	3.3	NR	NR	NR	9	49.6(41.9-57.3)	18.6(11.6-25.7)	6.1(2.6-9.6)	NR	NR	NR
**Ntekim,[33] 2019 (Ntekim et al., 2019), Nigeria**	32	Female	2008-2012	5	44.6(10.3)	NR	52	51.4	NR	77.0(61.7-92.3)	65.0(46.9 -83.1)	59.0(40.5-77.5)	53.0 (34.8-71.2)	NR
**Okobia,[34] 2001 (Okobia and Osime, 2001), Nigeria**	77	Both	1987-1996	10	38	Medical record	NR	NR	NR	NR	NR	NR	8.7 (3.6-13.8)	NR
**Olasehinde,[35] 2021 (Olasehinde et al., 2021), Nigeria**	472	Both	2010-2020	10	49.8 (12.2)	Medical record and verbal autopsy	20.5	NR	NR	NR	NR	NR	43.6 (37.7-49.5)	NR
**Agodirin,[1] 2015 (Agodirin et al., 2015), Nigeria**	56	Female	2010-2014	3.5	47.7 (11.7)	NR	NR	17.5(14.67-20.33)	64.3(51.4-77.2)	26.8(16.5-37.1)	5.3(1.9-8.7)	NR	NR	NR

© 2024 Limenih MA et al. *JAMA Network Open*.

**Otu,[36] 1989 (Otu et al., 1989), Nigeria**	67	Both	1980-1984	5	40	NR	NR	NR	NR	NR	NR	NR	14.0(6.2-21.8)	NR
**Parag,[37] 2016 (Parag and Buccimazza, 2016), South Africa**	32	Female	2008-2012	5	NR	Verbal autopsy	NR	NR	NR	NR	NR	NR	65.6 (47.2-84.1)	NR
**Plessis,[16] 2015 (Du Plessis and Apffelstaedt, 2015), South Africa**	237	Female	2003-2008	5	56	NR	NR	NR	NR	NR	NR	NR	44.5 (33.5-55.5)	NR
**Schulz,[17] 2018 (Eber-Schulz et al., 2018), Ethiopia**	107	Female	2010-2016	6	45(Range ;16-38)	Medical record and verbal autopsy	28	16.7	76.3(69.1-83.5)	53.2 (42.2-64.4)	35.2(25.9 -44.6)	27.6(19.3-35.9)	20.2 (12.6-27.8)	NR
**Shibabaw,[38] 2019 (Shibabaw et al., 2019), Ethiopia**	627	Female	2012-2017	6	42.6 (12.2)	Medical record	NR	56.5(53.4-60.8)	97.2(95.8-98)	89.8 (87.5-92.1)	80.0(77-84)	66.3(58.6-68)	46 (41.2-50.8)	NR
**Somé,[39] 2022 (Some et al., 2022), Burkina Faso**	368	Female	2015-2021	6	46.6 (12.1)	NR	23.9	36	NR	NR	50(43.5-56.5)	NR	22 (16.5-27.5)	NR
**Ssentongo,[40] 2022 (Ssentongo et al., 2022), Ghana**	129	Female	2014-2018	3	51 (IQR;45)	Medical record and verbal autopsy	NR	40(20-60)	NR	NR	52(42.3-61.7)	NR	NR	NR
**Tiruneh,[42] 2021 (Tiruneh et al., 2021), Ethiopia**	300	Female	2015-2020	5	61 (IQR;56)	Medical record and verbal autopsy	15	NR	NR	54.2(49.1-66.3)	NR	NR	25.8 (22.2-36.9)	NR
**Traore,[43] 2020 (Traore et al., 2022), Guinea**	210	Both	2007-2016	9	47.5 (13)	NR	NR	NR	NR	NR	NR	NR	49.5 (40-60)	NR
**Wambua,[44] 2022 (Wambua et al., 2022), Kenya**	116	Female	2020-2021	5	56 (15.8)	NR	NR	NR	82.2(75.8-88.6)	71.6(63.4-79.8)	69(60.9-77.1)	64.7(56-73.4)	63 (54.7-71.3)	NR
**Wuraola,[45] 2022 (Wuraola et al., 2022), Nigeria**	87	Both	2010-2018	9	71 (6.6)	NR	NR	28.3	80.0(71.6-88.4)	53.0(41.3-64.7)	45(33.2-56.8)	37.0(26.1-47.6)	42(30.8-53.2)	NR
**Youngblood,[46] 2020 (Youngblood et al., 2020), Malawi**	100	Female	2016-2018	2	NR	NR	NR	20.2	74.0(62-83)	NR	NR	NR	NR	NR
**Zingue,[47] 2021 (Zingue et al., 2021), Cameroon**	344	Female	2010-2015	5	45.4 (13.4)	Medical record and verbal autopsy	NR	38.3	87.7(84.8-90.6)	64.0(58.1-69.9)	54(48.1-59.9)	46.0(39.6-52.4)	40(33.5-46.5)	NR
**Zongo,[48] 2022 (Zongo et al., 2022), Burkina Faso**	550	Female	2013-2020	5	47.9 (12.6)	verbal autopsy	NR	NR	NR	NR	NR	NR	66.2(61.1-74.5)	NR
**Zongo,[49] 2018 (Zongo et al., 2018), Burkina Faso**	51	Male	1990-2015	5	60.9 (8.4)	Medical record and verbal autopsy	40.8	NR	97(76.5-99.8)	96(78.6-99.5)	84(58.8-86.6)	59(39.2-64.7)	49.0(41.2-60.8)	NR

CI: Confidence Interval; FU: Follow-up; IQR: Interquartile Range; NR: Not reported; SD: Standard Deviation

© 2024 Limenih MA et al. *JAMA Network Open*.

eFigure 1. Risk of Bias of Included Studies by Study Period Based on the Newcastle-Ottawa Scale

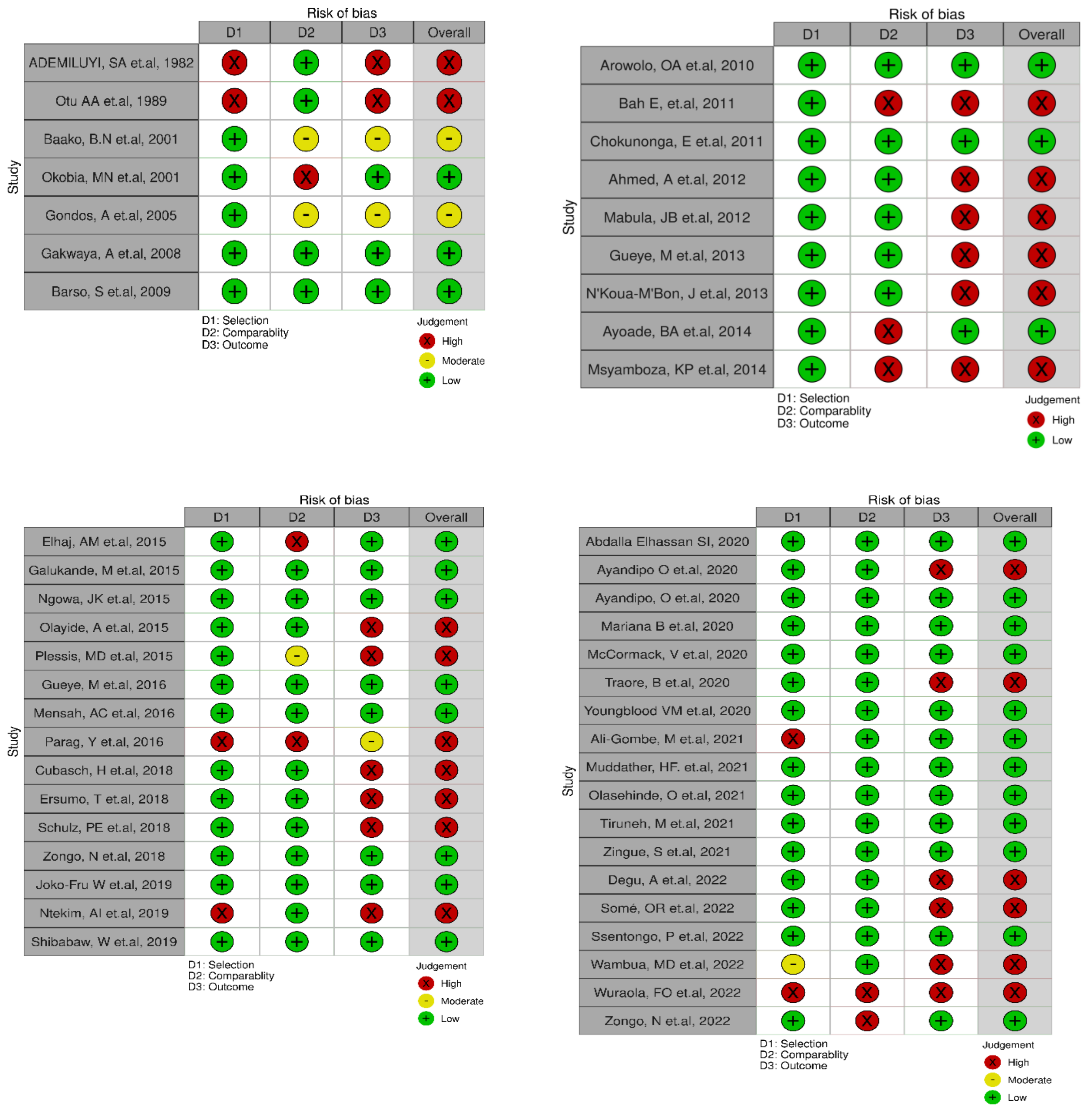

© 2024 Limenih MA et al. *JAMA Network Open*.

eFigure 2. Forest Plots Indicating the 2-Year and 4-Year Survival Rates of Patients With Breast Cancer in Sub-Saharan Africa

a)

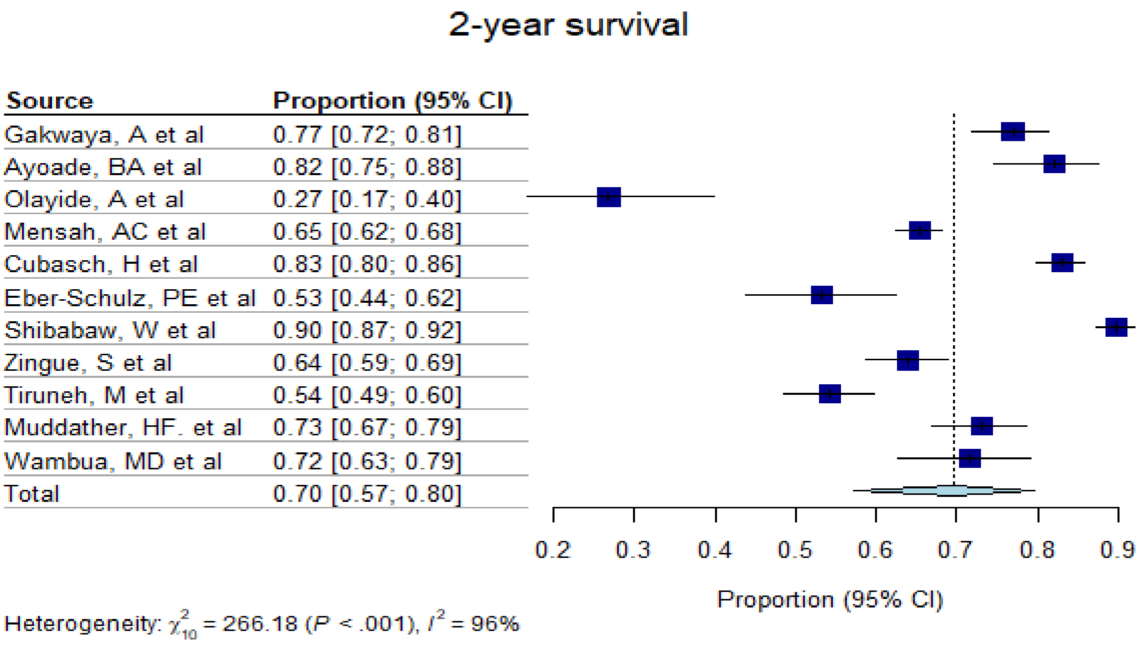

b)

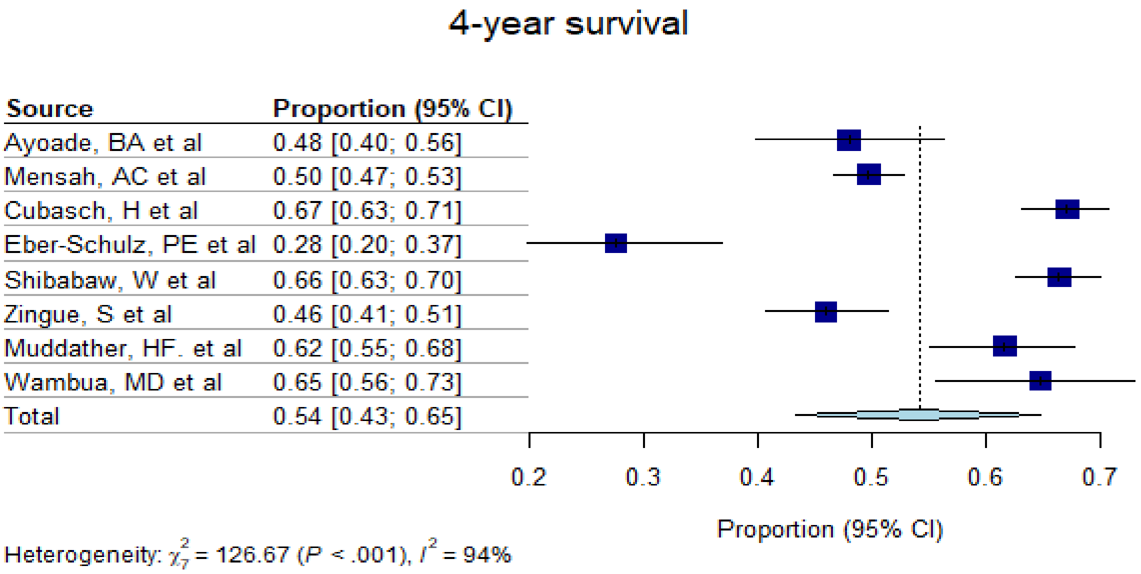

© 2024 Limenih MA et al. *JAMA Network Open*.

eFigure 3. Forest Plot Indicating the 5-Year Survival Rates of Breast Cancer Among Male Patients in Sub-Saharan Africa

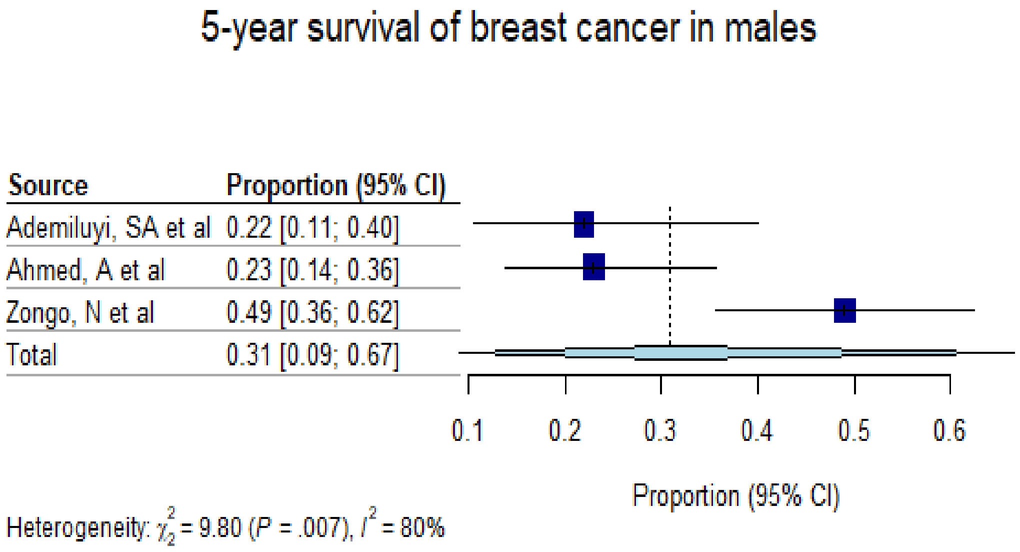

© 2024 Limenih MA et al. *JAMA Network Open*.

eFigure 4. Forest Plots Indicating Subgroup Analysis by Human Development Index, Study Period and Study Quality

A)

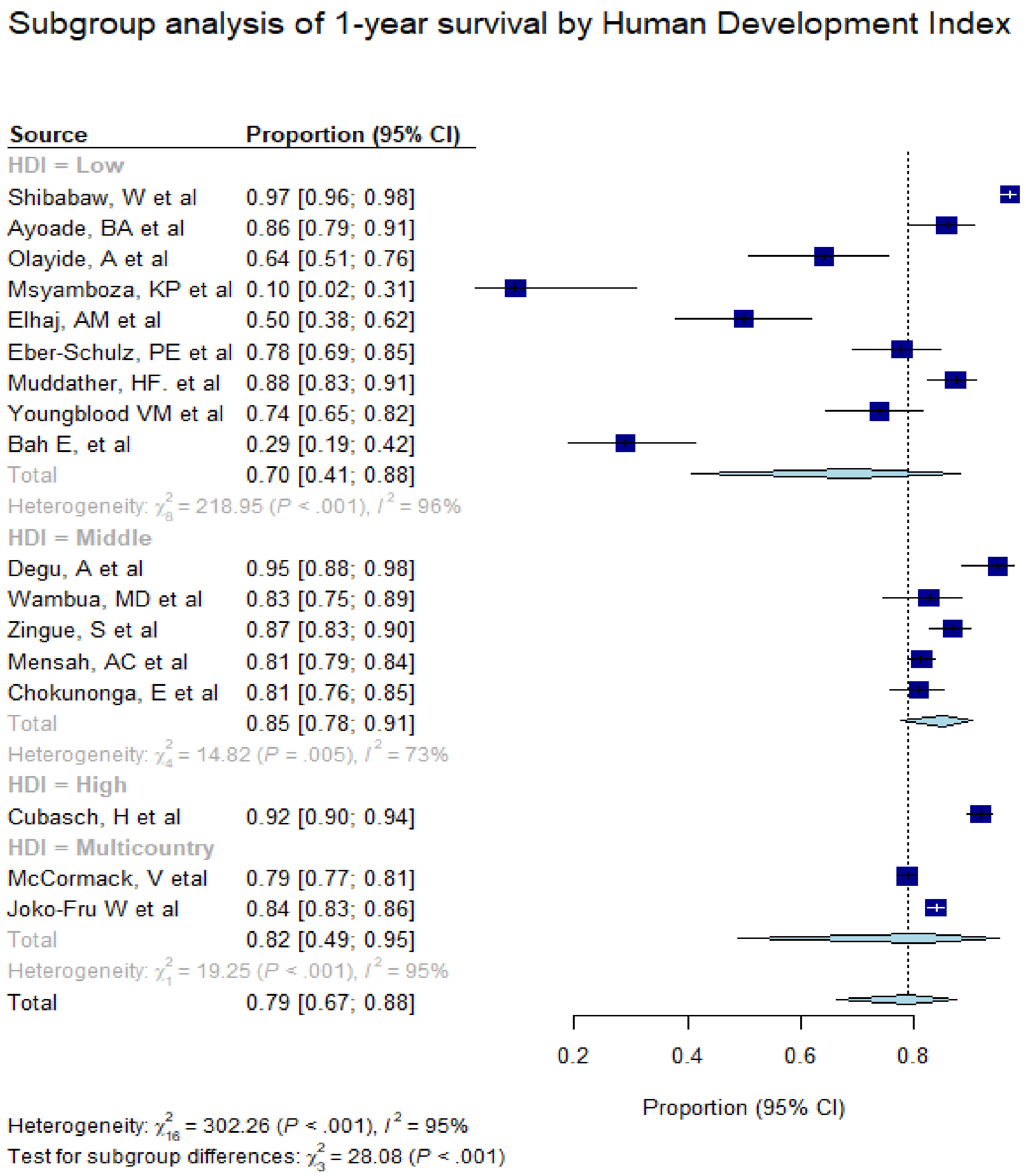

© 2024 Limenih MA et al. *JAMA Network Open*.

B)

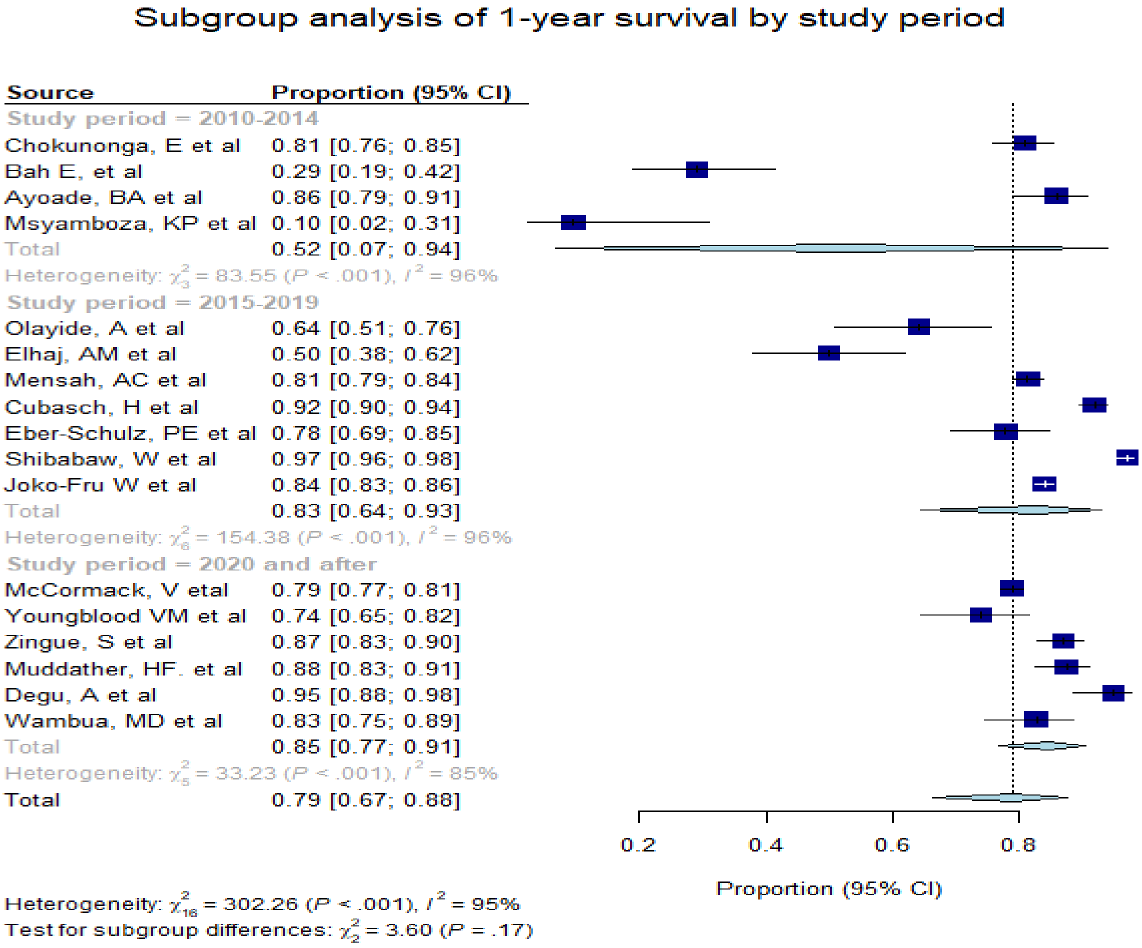

© 2024 Limenih MA et al. *JAMA Network Open*.

C)

Subgroup analysis of 1-year survival by study quality

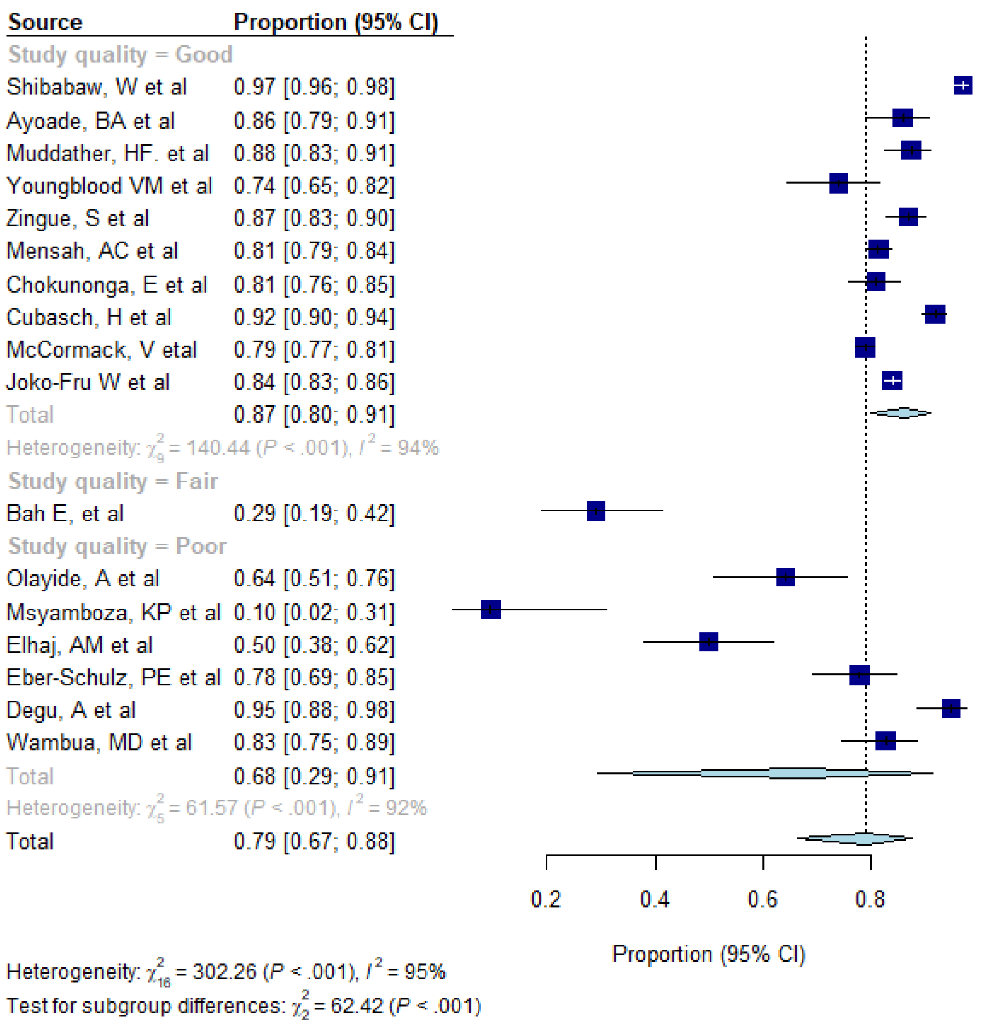

© 2024 Limenih MA et al. *JAMA Network Open*.

D)

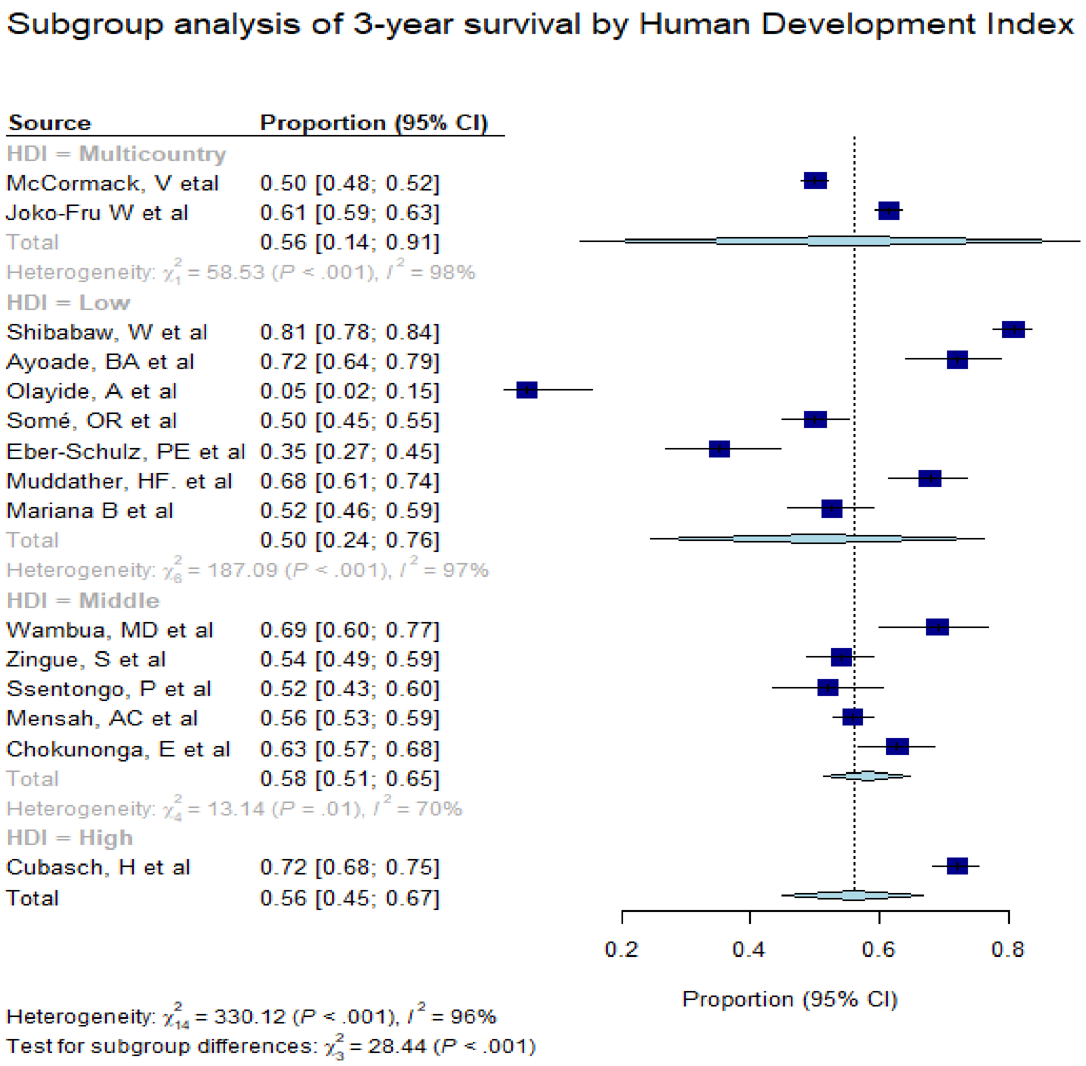

© 2024 Limenih MA et al. *JAMA Network Open*.

E)

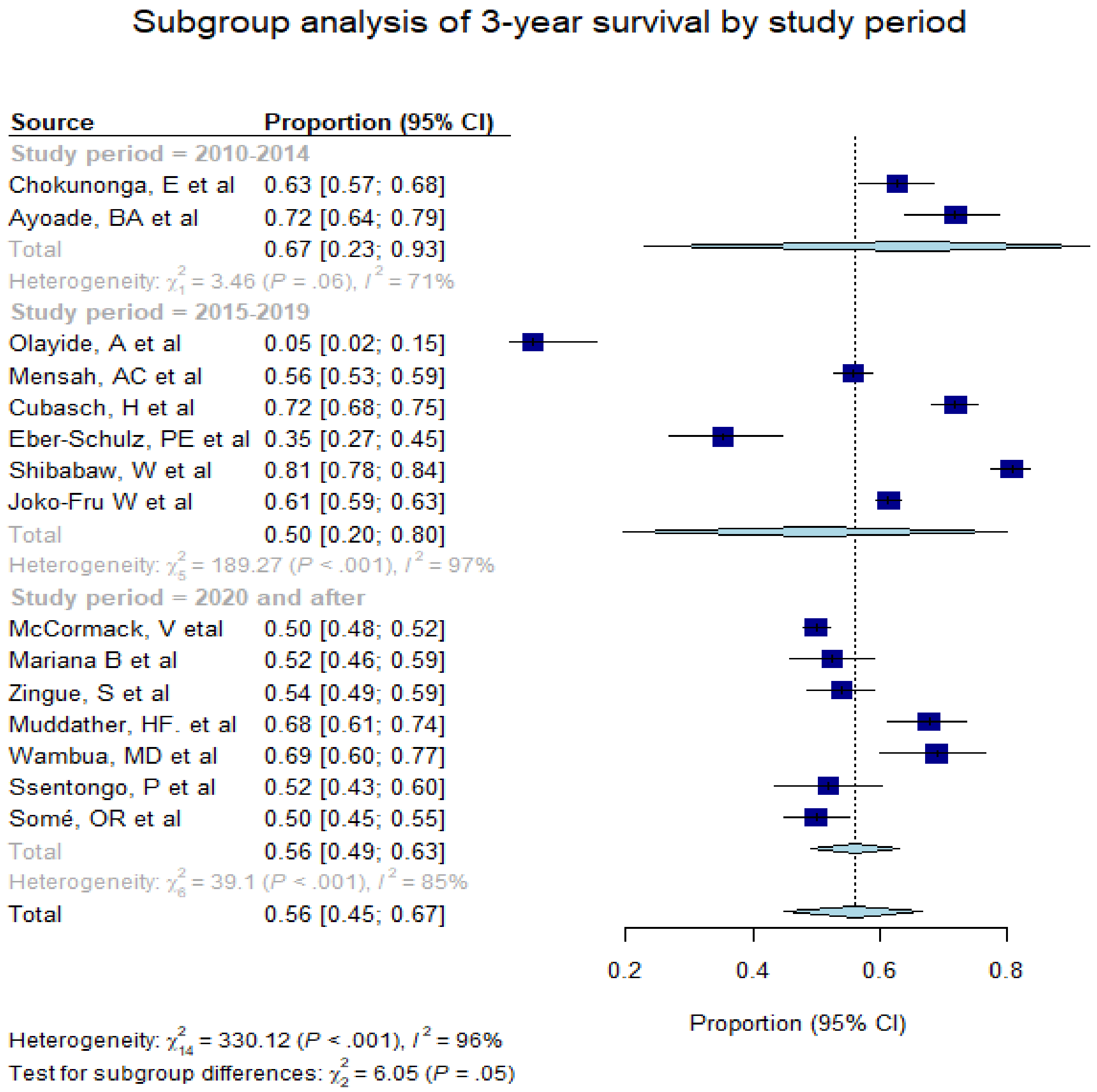

© 2024 Limenih MA et al. *JAMA Network Open*.

F)

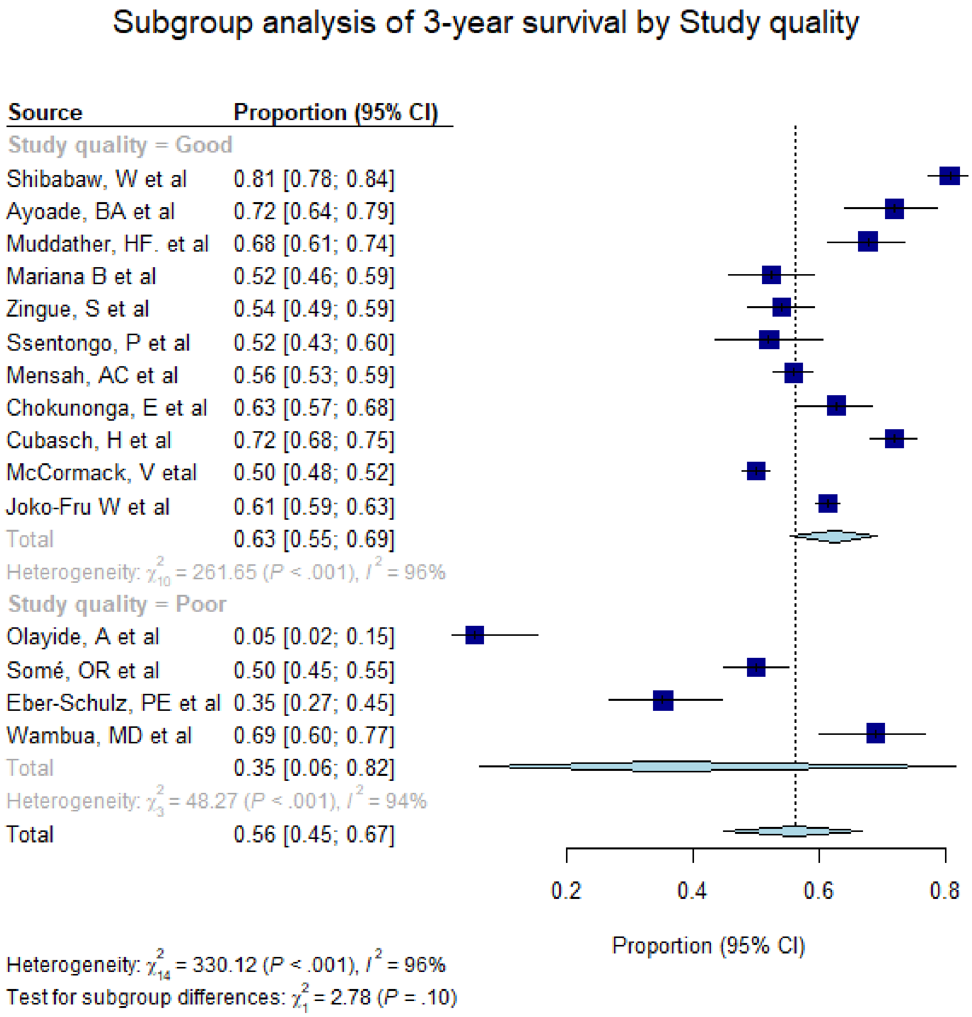

© 2024 Limenih MA et al. *JAMA Network Open*.

G)

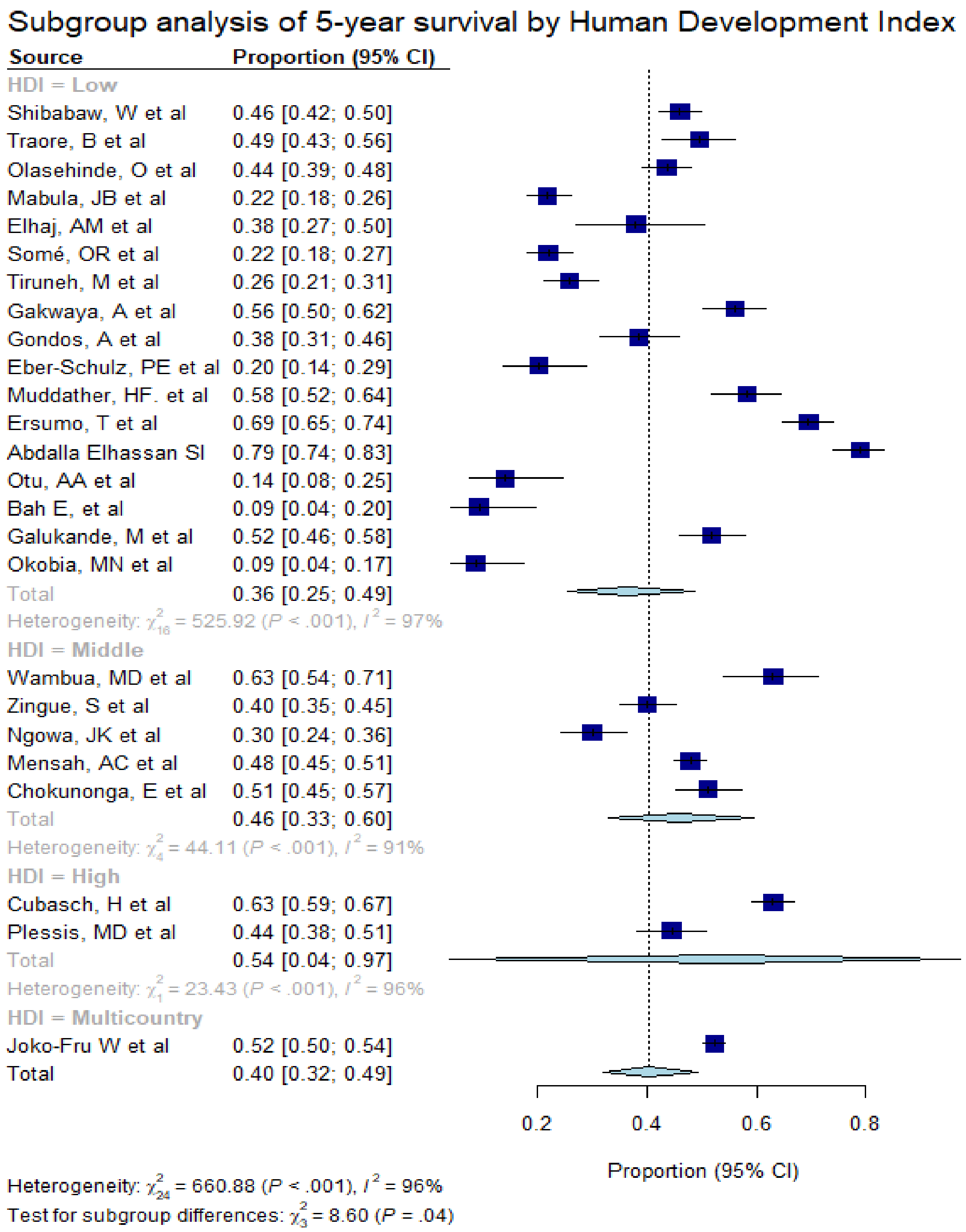

© 2024 Limenih MA et al. *JAMA Network Open*.

H)

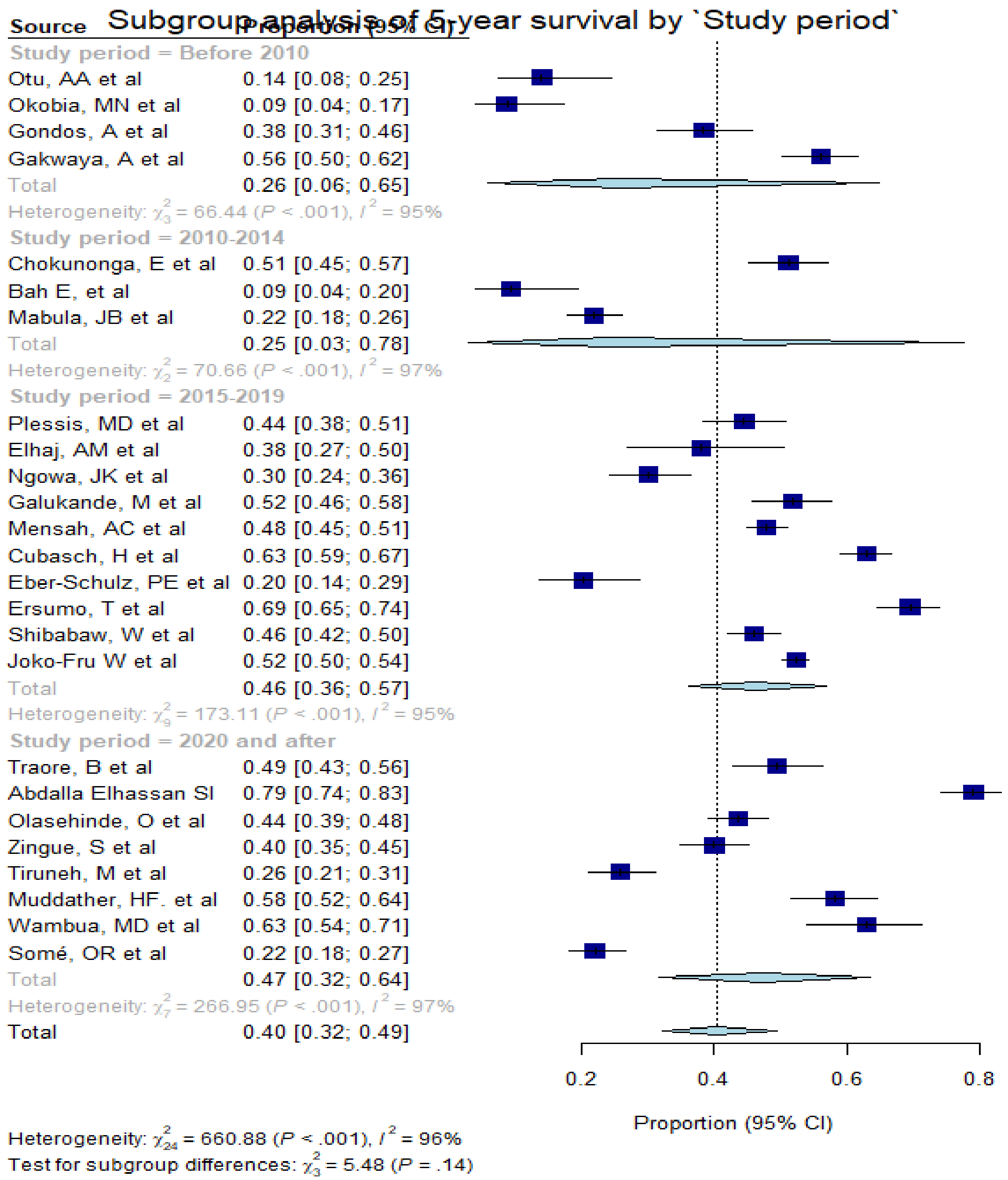

© 2024 Limenih MA et al. *JAMA Network Open*.

I)

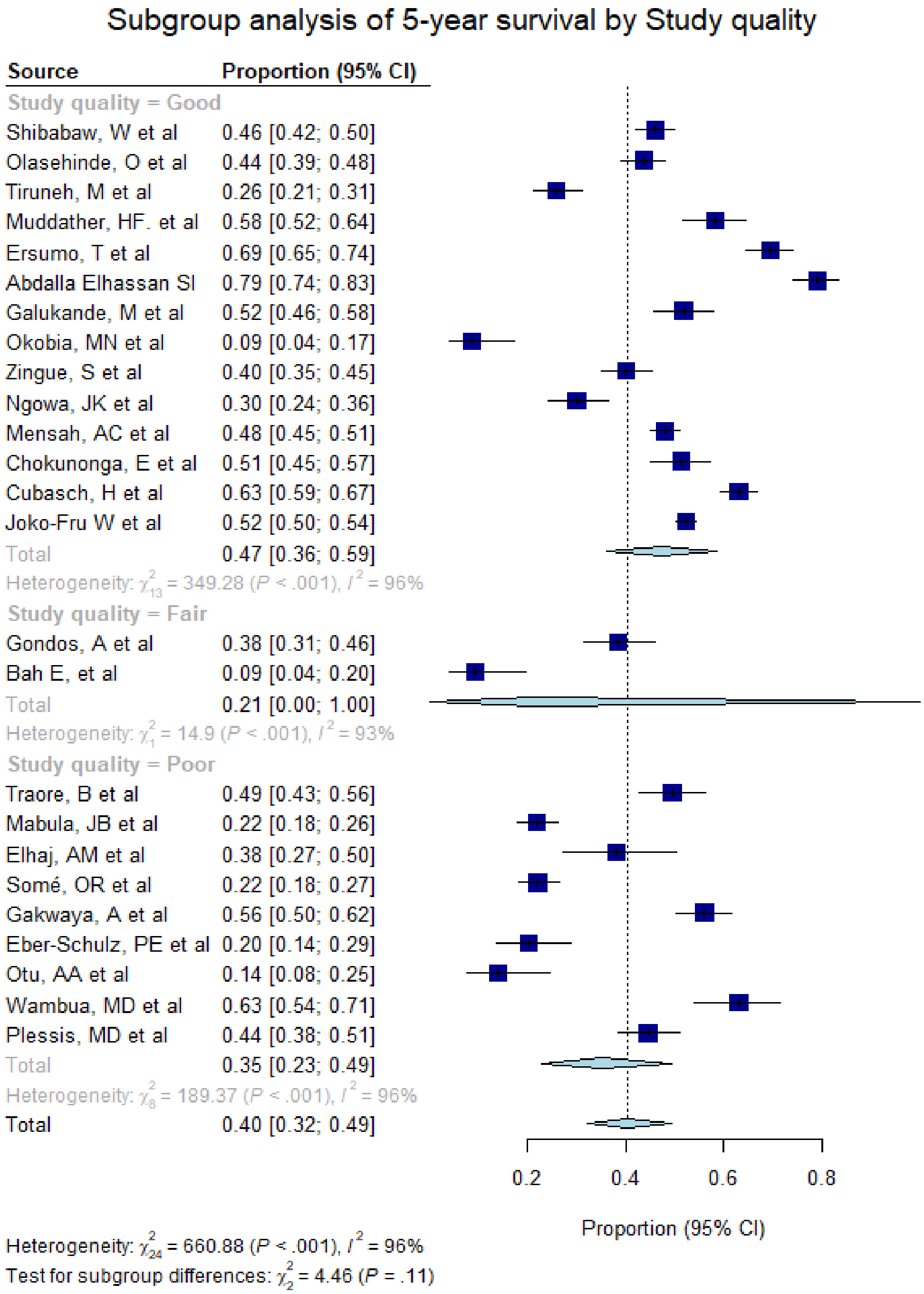

© 2024 Limenih MA et al. *JAMA Network Open*.

eFigure 5. Funnel Plots Indicating Publication Bias Assessment by Study Year

A)

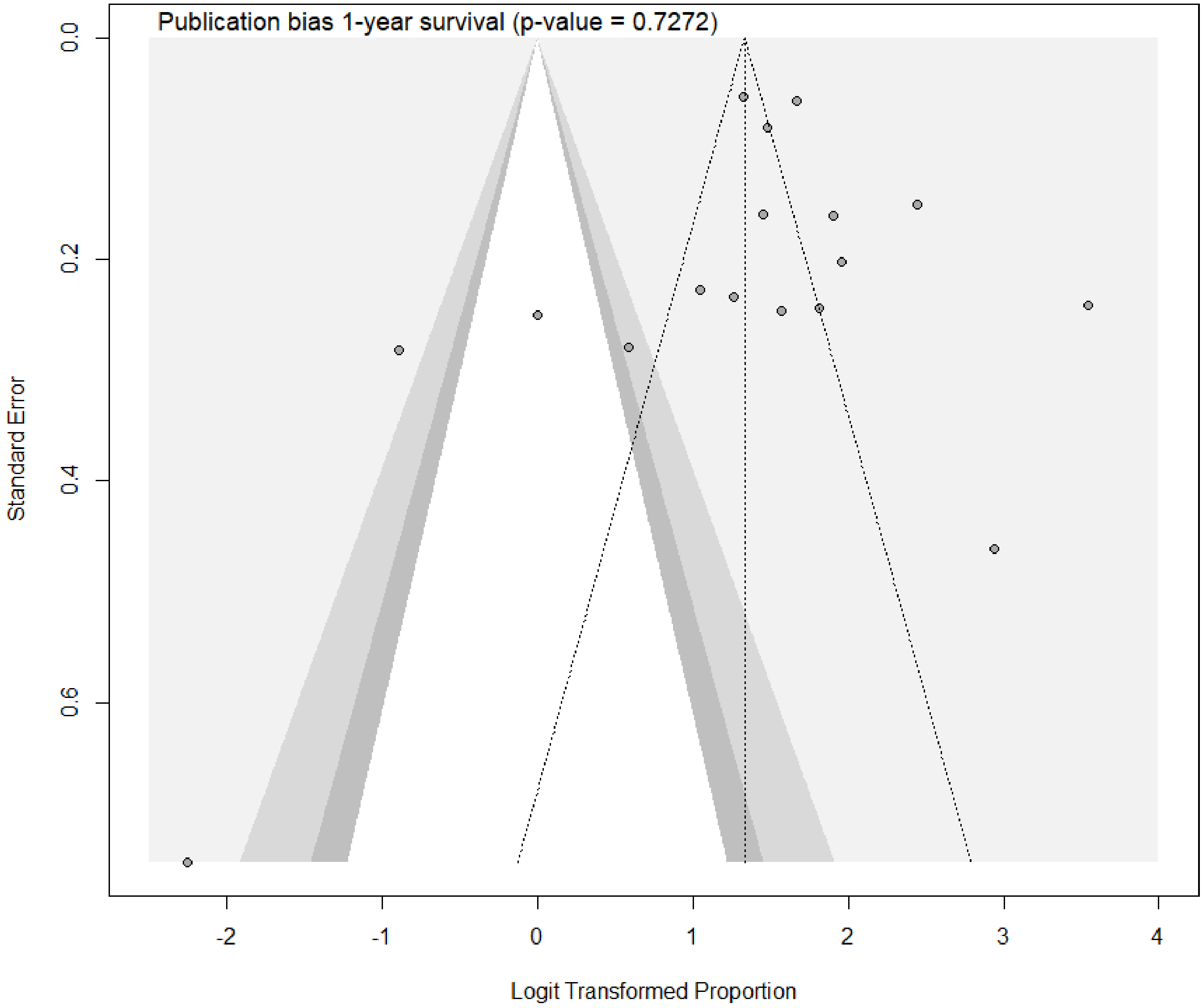

© 2024 Limenih MA et al. *JAMA Network Open*.

B)

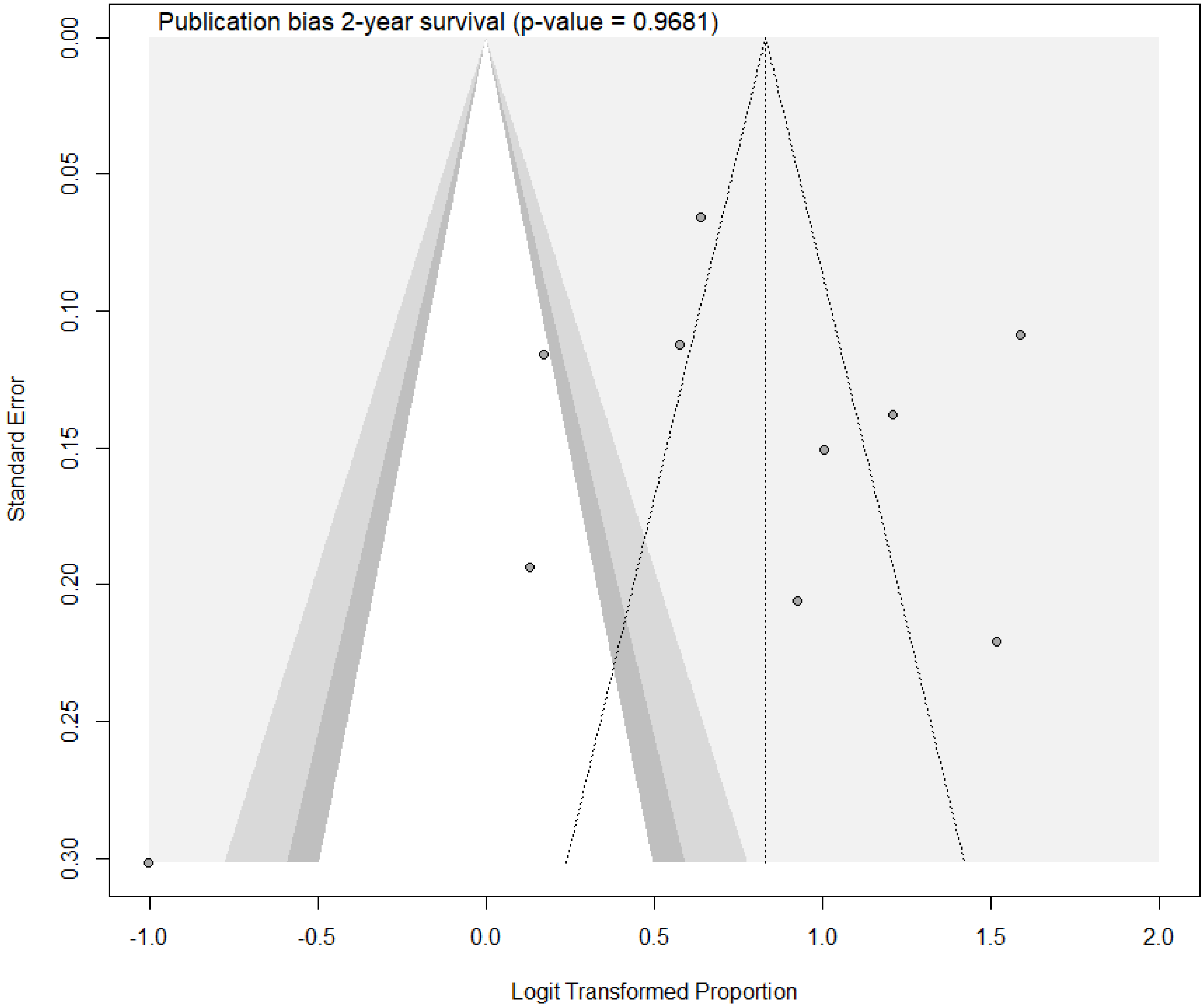

© 2024 Limenih MA et al. *JAMA Network Open*.

C)

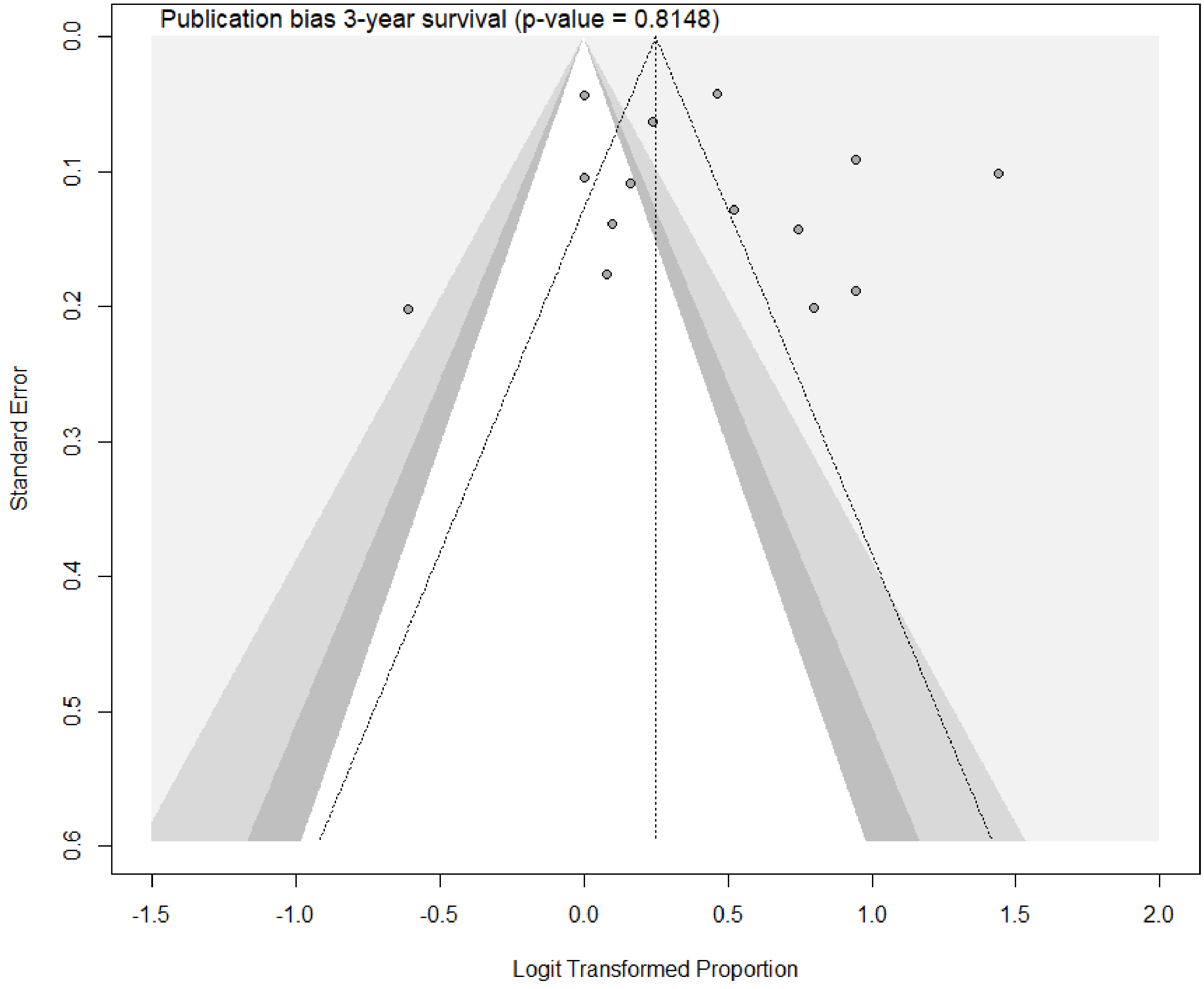

© 2024 Limenih MA et al. *JAMA Network Open*.

D)

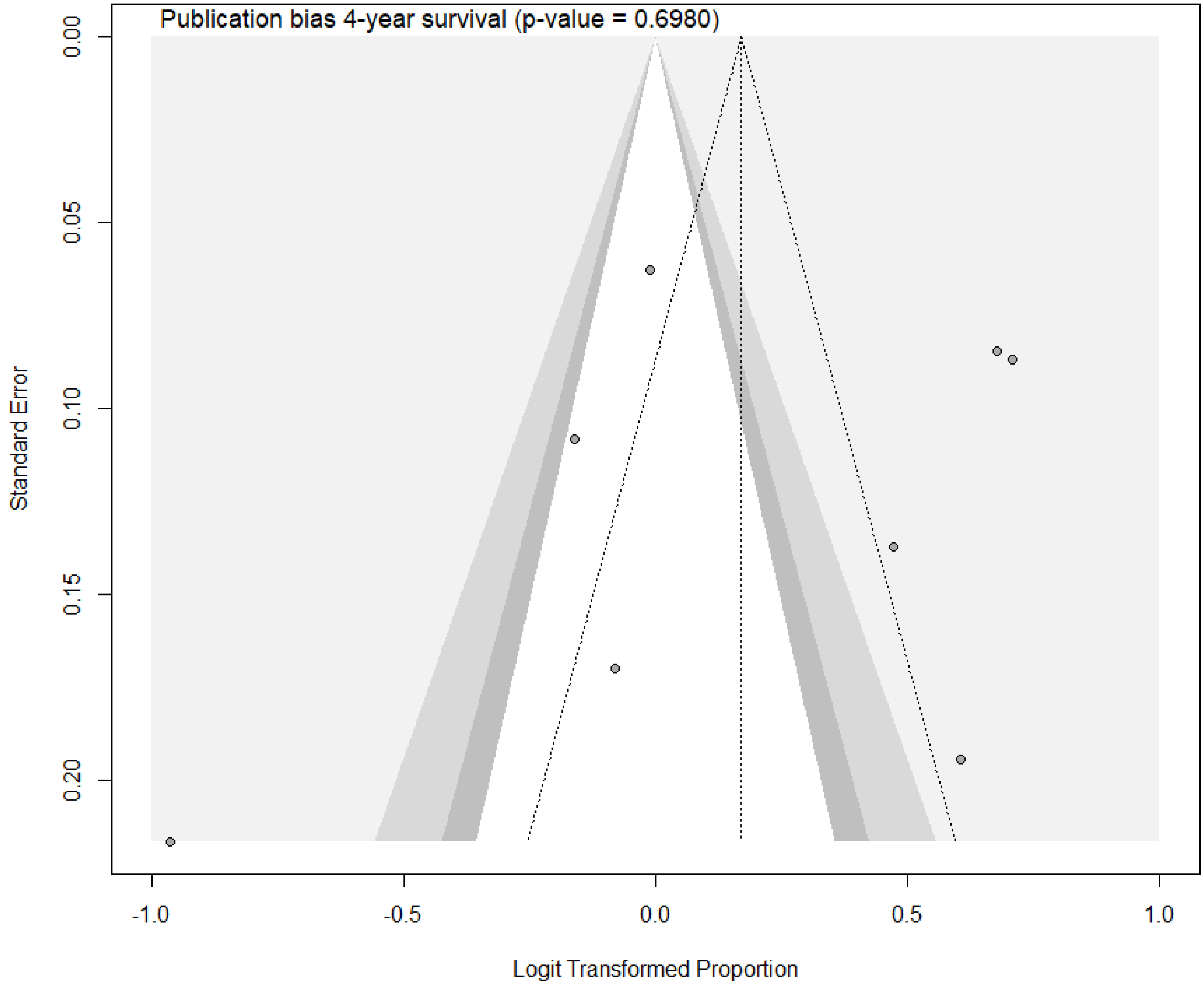

© 2024 Limenih MA et al. *JAMA Network Open*.

E)

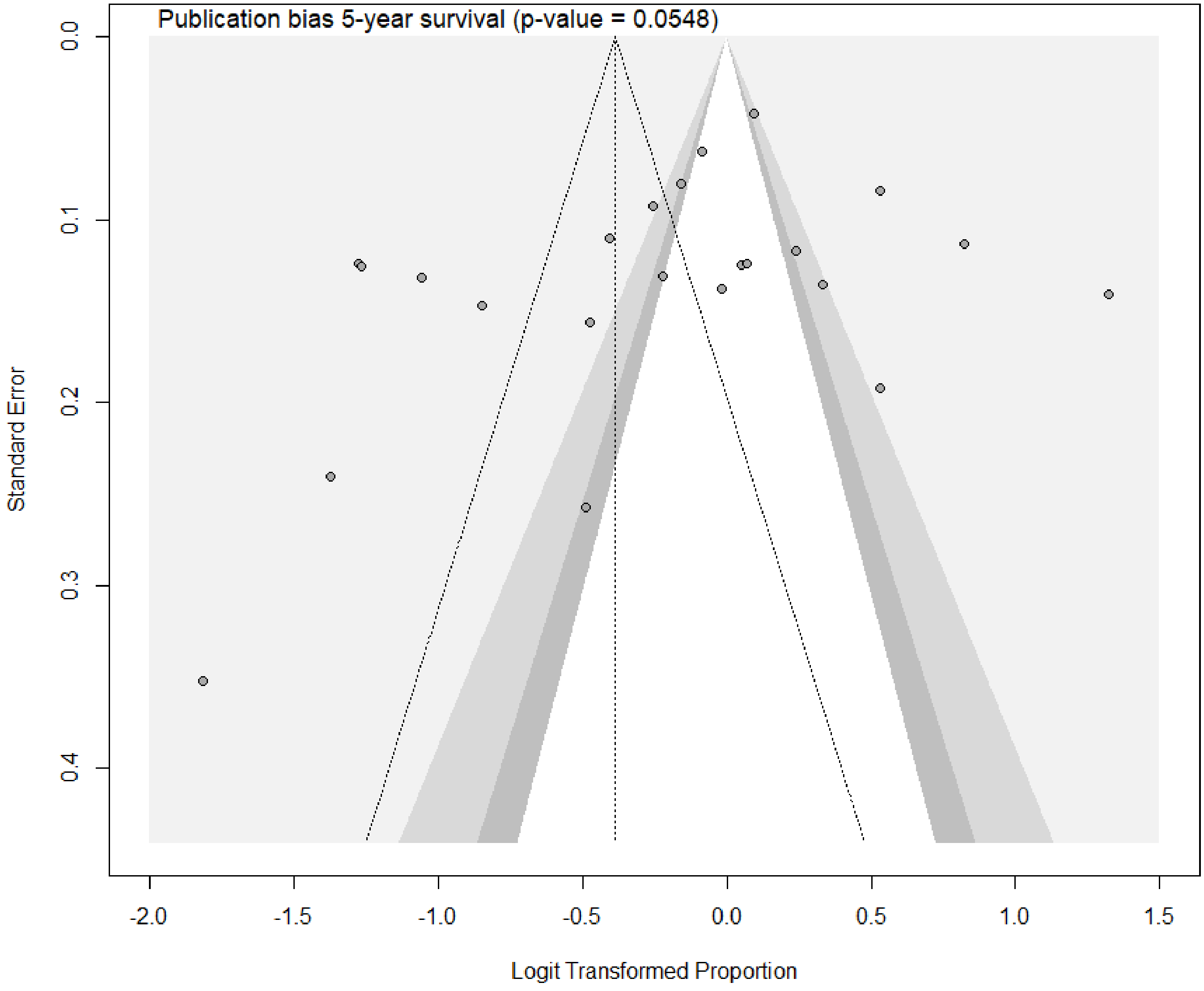

© 2024 Limenih MA et al. *JAMA Network Open*.

eReferences

1. AGODIRIN, O., OLATOKE, S., RAHMAN, G., ADEOTI, M., OYEYEMI, G., DUROJAIYE, A., KOLAWOLE, D. & AGBOOLA, J. 2015. How effective is the treatment of locally advanced and metastatic breast cancer in developing centres?: A retrospective review. *Ethiopian journal of health sciences,* 25**,** 337-344.
2. AHMED, A., UKWENYA, Y., ABDULLAHI, A. & MUHAMMAD, I. 2012. Management and Outcomes of Male Breast Cancer in Zaria, Nigeria. *International Journal of Breast Cancer,* 2012**,** 845143.
3. AJAYI, D. O. S., OSEGBE, D. N. & ADEMILUYI, S. A. 1982. Carcinoma of the male breast in west Africans and a review of world literature. *Cancer,* 50**,** 1664-1667.
4. ALI-GOMBE, M., MUSTAPHA, M. I., FOLASIRE, A., NTEKIM, A. & CAMPBELL, O. B. 2021. Pattern of survival of breast cancer patients in a tertiary hospital in South West Nigeria. *ecancermedicalscience,* 15.
5. AROWOLO, O. A., AKINKUOLIE, A. A., LAWAL, O. O., ALATISE, O. I., SALAKO, A. A. & ADISA, A. O. 2010. The Impact of Neoadjuvant Chemotherapy on Patients with Locally Advanced Breast Cancer in a Nigerian Semiurban Teaching Hospital: A Single-center Descriptive Study. *World Journal of Surgery,* 34**,** 1771-1778.
6. AYANDIPO, O. O., AFUWAPE, O. O., ADEPOJU, O. J., AJIBOYE, J. A. & OGUNDIRAN, T. O. 2020a. Stage-specific five-year survival outcomes in women treated for early stage breast cancer in Ibadan, Nigeria. *Nigerian Journal of Medicine,* 29**,** 152-157.
7. AYANDIPO, O. O., OGUN, G. O., ADEPOJU, O. J., FATUNLA, E. O., AFOLABI, A. O., OSUALA, P. C. & OGUNDIRAN, T. O. 2020b. Impact of axillary node-positivity and surgical resection margins on survival of women treated for breast cancer in Ibadan, Nigeria. *Ecancermedicalscience,* 14**,** 1084.
8. AYOADE, B., AGBOOLA, A., OLATUNJI, A., TADE, A., SALAMI, B. & ADEKOYA, A. 2014. Clinical characteristics and survival outcome of breast cancer in Southwest Nigerian women. *Journal Africain du Cancer/African Journal of Cancer,* 2**,** 79-84.
9. BAAKO, B. & BADOE, E. 2001. Treatment of breast cancer in Accra: 5-year survival. *Survival,* 2**,** 5years.
10. BAH, E., SAM, O., WHITTLE, H., RAMANAKUMAR, A. & SANKARANARAYANAN, R. 2011. Cancer survival in the Gambia, 1993-1997. *IARC scientific publications***,** 97-100.
11. BASRO, S. & APFFELSTAEDT, J. P. 2010. Breast cancer in young women in a limited-resource environment. *World journal of surgery,* 34**,** 1427-1433.
12. BRANDÃO, M., GUISSEVE, A., BATA, G., ALBERTO, M., FERRO, J., GARCIA, C., ZAQUEU, C., LORENZONI, C., LEITÃO, D. & COME, J. 2020. Breast cancer subtypes: implications for the treatment and survival of patients in Africa—a prospective cohort study from Mozambique. *ESMO open,* 5**,** e000829.
13. CHOKUNONGA, E., BOROK, M., CHIRENJE, Z., NYABAKAU, A. & PARKIN, D. 2011. Cancer survival in Harare, Zimbabwe, 1993Γ1997.
14. CUBASCH, H., DICKENS, C., JOFFE, M., DUARTE, R., MURUGAN, N., CHIH, M. T., MOODLEY, K., SHARMA, V., AYENI, O. & JACOBSON, J. S. 2018. Breast cancer survival in Soweto, Johannesburg, South Africa: a receptor-defined cohort of women diagnosed from 2009 to 11. *Cancer epidemiology,* 52**,** 120-127.
15. DEGU, A., TEREFE, E. M., SOME, E. S. & TEGEGNE, G. T. 2022. Treatment Outcomes and Its Associated Factors Among Adult Patients with Selected Solid Malignancies at Kenyatta National Hospital: A Hospital-Based Prospective Cohort Study. *Cancer Management and Research***,** 1525-1540.

© 2024 Limenih MA et al. *JAMA Network Open*.

16. DU PLESSIS, M. & APFFELSTAEDT, J. P. 2015. Treatment outcomes of breast carcinoma in a resource-limited environment surgery. *South African Journal of Surgery,* 53**,** 43-47.
17. EBER-SCHULZ, P., TARIKU, W., REIBOLD, C., ADDISSIE, A., WICKENHAUSER, C., FATHKE, C., HAUPTMANN, S., JEMAL, A., THOMSSEN, C. & KANTELHARDT, E. J. 2018. Survival of breast cancer patients in rural Ethiopia. *Breast cancer research and treatment,* 170**,** 111-118.
18. ELHAJ, A. M., ABDALSALAM, A., ABUIDRIS, A. & ELTAYEB, A. 2015. Overall survival of females with breast cancer in the National Cancer Institute, University of Gezira, Sudan. *Sudan Medical Monitor,* 10**,** 1.
19. ELHASSAN, S. I. A. 2020. The five-year survival rate of breast cancer at Radiation and Isotopes Centre Khartoum, Sudan. *Heliyon,* 6.
20. GAKWAYA, A., KIGULA-MUGAMBE, J., KAVUMA, A., LUWAGA, A., FUALAL, J., JOMBWE, J., GALUKANDE, M. & KANYIKE, D. 2008. Cancer of the breast: 5-year survival in a tertiary hospital in Uganda. *British journal of cancer,* 99**,** 63-67.
21. GALUKANDE, M., WABINGA, H. & MIREMBE, F. 2015. Breast cancer survival experiences at a tertiary hospital in sub-Saharan Africa: a cohort study. *World journal of surgical oncology,* 13**,** 1-8.
22. GONDOS, A., BRENNER, H., WABINGA, H. & PARKIN, D. 2005. Cancer survival in kampala, Uganda. *British journal of cancer,* 92**,** 1808-1812.
23. GUÈYE, M., GUÈYE, S., MBAYE, M., NIASSE DIA, F., FAYE DIÉMÉ, M., NIANG, M., DIOUF, A. & MOREAU, J.-C. 2013. Clinical features and prognosis of triple negative breast cancer at the senology unit of Aristide-Le-Dantec teaching hospital. *Journal Africain du Cancer/African Journal of Cancer,* 5**,** 42-47.
24. GUEYE, M., KANE GUEYE, S. M., NDIAYE GUEYE, M. D., NIASSE DIA, F., GASSAMA, O., DIALLO, M. & MOREAU, J. C. 2016. Breast cancer in women younger than 35 years : features and outcomes in the breast unit at Aristide le Dantec Teaching Hospital, Dakar. *Med Sante Trop,* 26**,** 377-381.
25. JOKO-FRU, W. Y., MIRANDA-FILHO, A., SOERJOMATARAM, I., EGUE, M., AKELE-AKPO, M.-T., N'DA, G., ASSEFA, M., BUZIBA, N., KORIR, A., KAMATE, B., TRAORE, C., MANRAJ, S., LORENZONI, C., CARRILHO, C., HANSEN, R., FINESSE, A., SOMDYALA, N., WABINGA, H., CHINGONZOH, T., BOROK, M., CHOKUNONGA, E., LIU, B., KANTELHARDT, E., MCGALE, P. & PARKIN, D. M. 2020. Breast cancer survival in sub-Saharan Africa by age, stage at diagnosis and human development index: A population-based registry study. *International Journal of Cancer,* 146**,** 1208-1218.
26. MABULA, J. B., MCHEMBE, M. D., CHALYA, P. L., GIITI, G., CHANDIKA, A. B., RAMBAU, P. F., MASALU, N. & GILYOMA, J. M. 2012. Stage at diagnosis, clinicopathological and treatment patterns of breast cancer at Bugando Medical Centre in north-western Tanzania. *Tanzania journal of health research,* 14.
27. MCCORMACK, V., MCKENZIE, F., FOERSTER, M., ZIETSMAN, A., GALUKANDE, M., ADISA, C., ANELE, A., PARHAM, G., PINDER, L. F. & CUBASCH, H. 2020. Breast cancer survival and survival gap apportionment in sub-Saharan Africa (ABC-DO): a prospective cohort study. *The Lancet Global health,* 8**,** e1203-e1212.
28. MENSAH, A. C. 2016. Survival outcomes of breast cancer in Ghana: An analysis of clinicopathological features.
29. MSYAMBOZA, K. P., MANDA, G., TEMBO, B., THAMBO, C., CHITETE, L., MINDIERA, C., FINCH, L. K. & HAMLING, K. 2014. Cancer survival in Malawi: a retrospective cohort study. *Pan African Medical Journal,* 19.
30. MUDDATHER, H. F., ELHASSAN, M. M. & FAGGAD, A. 2021. Survival outcomes of breast cancer in sudanese women: a hospital-based study. *JCO Global Oncology,* 7**,** 324-332.

© 2024 Limenih MA et al. *JAMA Network Open*.

31. N'KOUA-M'BON, J. B., BAMBARA, A. T., MOUKASSA, D. & GOMBÉ-MBALAWA, C. 2013. [Clinical and outcome characteristics of inflammatory breast cancers in Brazzaville]. *Bull Cancer,* 100**,** 147-53.
32. NGOWA, J. D. K., KASIA, J. M., YOMI, J., NANA, A. N., NGASSAM, A., DOMKAM, I., SANDO, Z. & NDOM, P. 2015. Breast cancer survival in cameroon: analysis of a cohort of 404 patients at the yaoundé general hospital. *Advances in Breast Cancer Research,* 4**,** 44.
33. NTEKIM, A. I., FOLASIRE, A. M. & ALI-GOMBE, M. 2019. Survival pattern of rare histological types of breast cancer in a Nigerian institution. *Pan African Medical Journal,* 34.
34. OKOBIA, M. & OSIME, U. 2001. Clinicopathological study of carcinoma of the breast in Benin City. *African journal of reproductive health,* 5**,** 56-62.
35. OLASEHINDE, O., ALATISE, O., OMISORE, A., WURAOLA, F., ODUJOKO, O., ROMANOFF, A., AKINKUOLIE, A., AROWOLO, O., ADISA, A. & KNAPP, G. 2021. Contemporary management of breast cancer in Nigeria: Insights from an institutional database. *International journal of cancer,* 148**,** 2906-2914.
36. OTU, A., EKANEM, I.-O., KHALIL, M., EKPO, D. & ATTAH, E. 1989. Characterization of breast cancer subgroups in an African population. *Journal of British Surgery,* 76**,** 182-184.
37. PARAG, Y. & BUCCIMAZZA, I. 2016. How long are elderly patients followed up with mammography after the diagnosis of breast cancer? A single-centre experience in a developing country. *South African Medical Journal,* 106**,** 721-723.
38. SHIBABAW, W., MULUGETA, T., ABERA, H., ASMARE, Y. & YIRGA, T. 2019. Survival status and predictors of mortality among Breast cancer patients at Black lion specialized hospital, Adult oncology unit, Addis Ababa, Ethiopia, 2018. A retrospective follow-up study with survival analysis. *bioRxiv***,** 636431.
39. SOME, O. R., BAGUE, A. H., KONKOBO, D., HIEN, D., DEMBELE, A., BELEMLILGA, G., KONSEGRE, V. & ZONGO, N. 2022. Breast Cancer in Bobo-Dioulasso, Burkina Faso: Management Outcomes. *ONCOLOGIE,* 24**,** 173-184.
40. SSENTONGO, P., OH, J. S., AMPONSAH-MANU, F., WONG, W., CANDELA, X., ACHARYA, Y., SSENTONGO, A. E. & DODGE, D. G. 2022. Breast cancer survival in eastern region of Ghana. *Frontiers in Public Health,* 10**,** 880789.
41. TESSEMA ERSUMO, M., GIRMAYE TAMRAT, M., BOGALE SOLOMON, M. & TARIKU GERO, M. 2018. BREAST CANCER IN A PRIVATE MEDICAL SERVICES CENTER: A 10-YEAR EXPERIENCE. *Age,* 35**,** 35-44.
42. TIRUNEH, M., TESFAW, A. & TESFA, D. 2021. Survival and Predictors of Mortality among Breast Cancer Patients in Northwest Ethiopia: A Retrospective Cohort Study. *Cancer Management and Research,* 13**,** 9225-9234.
43. TRAORE, B., KEITA, M., TOURE, A., CAMARA, I., BARRY, A. & KOULIBALY, M. 2022. Impact of surgery associated with radiotherapy on the prognosis of breast cancer–Guinea Breast Cancer Cohort Study. *Cancer Reports,* 5**,** e1554.
44. WAMBUA, M. D., DEGU, A. & TEGEGNE, G. T. 2022. Treatment outcomes and its associated factors among breast cancer patients at Kitui Referral Hospital. *SAGE Open Medicine,* 10**,** 20503121211067857.
45. WURAOLA, F. O., OLASEHINDE, O., DI BERNARDO, M., AKINKUOLIE, A. A., ADISA, A. O., ADEROUNMU, A. A., MOHAMMED, T. O., OMOYIOLA, O. Z., KINGHAM, T. P. & ALATISE, O. I. 2022. Breast cancer in elderly patients: a clinicopathological review of a Nigerian database. *ecancermedicalscience,* 16.
46. YOUNGBLOOD, V. M., NYIRENDA, R., NYASOSELA, R., ZUZE, T., YANG, Y., KUDOWA, E., MOSES, A., KINCAID, J., KAJOMBO, C. & KAMPANI, C. 2020. Outcomes and prognostic factors for women with breast cancer in Malawi. *Cancer Causes & Control,* 31**,** 393-402.

© 2024 Limenih MA et al. *JAMA Network Open*.

47. ZINGUE, S., ATENGUENA, E. O., ZINGUE, L. L., TUECHE, A. B., NJAMEN, D., NKOUM, A. B. & NDOM, P. 2021. Epidemiological and clinical profile, and survival of patients followed for breast cancer between 2010 and 2015 at the Yaounde General Hospital, Cameroon. *Pan African Medical Journal,* 39.
48. ZONGO, N., OUÉDRAOGO, S., BADO, C., KABORÉ, A. & DEM, A. 2022. Survival of patients operated on for breast cancer in Ouagadougou/Burkina Faso. *European Journal of Surgical Oncology,* 48**,** 2378-2384.
49. ZONGO, N., OUÉDRAOGO, S., KORSAGA-SOMÉ, N., SOMÉ, O. R., GO, N., OUANGRÉ, E., ZIDA, M., BONKOUNGOU, G., OUÉDRAOGO, A. S. & BAMBARA, A. H. 2018. Male breast cancer: diagnosis stages, treatment and survival in a country with limited resources (Burkina Faso). *World journal of surgical oncology,* 16**,** 1-7.

© 2024 Limenih MA et al. *JAMA Network Open*.
