# Supplementary material for: Survival Patterns Among Patients With Breast Cancer in Sub-Saharan Africa: A Systematic Review and Meta-Analysis
Source: JAMA Netw Open. 2024 May 14;7(5):e2410260. doi: 10.1001/jamanetworkopen.2024.10260 (PMC11094564; doi:10.1001/jamanetworkopen.2024.10260)
Supplement: Supplement 2. — Data Sharing Statement [file jamanetwopen-e2410260-s002.pdf]

Data Sharing Statement

Limenih. Survival Patterns Among Patients With Breast Cancer in Sub-Saharan Africa. *JAMA Netw Open*. Published May 14, 2024. doi:10.1001/jamanetworkopen.2024.10260

Data
Data available: No

Additional Information
Explanation for why data not available: It is NA: This is a meta-analysis study and all additional information and procedures will be uploaded to supplements
